# Photochemistry of iron-containing secondary organic aerosol is impacted by relative humidity during formation

**DOI:** 10.1038/s41612-025-01109-6

**Published:** 2025-07-01

**Authors:** Natasha M. Garner, Fabian Mahrt, Jens Top, Virginia Tadei, Kevin Kilchhofer, Satoshi Takahama, Imad El Haddad, David M. Bell, Markus Ammann, Peter A. Alpert

**Affiliations:** 1 PSI Center for Energy and Environmental Sciences, Paul Scherrer Institute, Villigen, Switzerland; 2 Department of Environmental System Science, Institute for Atmospheric and Climate Science, ETH Zurich, Zurich, Switzerland; 3 Laboratory of Atmospheric Processes and their Impacts, School of Architecture, Civil & Environmental Engineering, Ecole Polytechnique Fédérale de Lausanne, Lausanne, Switzerland; 4 Laboratory of Environmental Spectrochemistry, School of Architecture, Civil & Environmental Engineering, Ecole Polytechnique Fédérale de Lausanne, Lausanne, Switzerland; 5 Present Address: School of Chemistry, University of Leeds, Leeds, United Kingdom; 6 Present Address: Department of Chemistry, Aarhus University, Aarhus, Denmark; 7 Present Address: Physikalisch-Meteorologisches Observatorium Davos, World Radiation Center (PMOD/WRC), Davos Dorf, Switzerland; 8 Present Address: XRnanotech AG, Villigen, Switzerland

**Keywords:** Atmospheric science, Atmospheric chemistry, Atmospheric chemistry

## Abstract

Secondary organic aerosol (SOA) comprises most of the submicron atmospheric particle mass, and often becomes internally mixed with other particles. When SOA mixes with transition metal (e.g., iron) containing particles, metal-organic complexes can form, enabling photochemical reactions that change aerosol physicochemical properties. We studied the photochemistry of α-pinene SOA formed on iron-containing ammonium sulfate seed particles at varying relative humidities (RH). Chemical composition and photochemical reduction of particles were analyzed by X-ray spectromicroscopy and infrared spectroscopy. SOA formed at low vs. high RH had different chemical functionality, including abundant carboxylic acids and alcohols. Following photolysis, carboxylic acids and unsubstituted alkanes decreased, and alcohols increased, consistent with decarboxylation reactions. Iron in SOA formed at high RH was readily photochemically reduced, but iron in SOA formed at low RH was not. Overall, RH conditions at SOA formation affect not only chemical composition but also iron-complex formation and hence photochemical processing of aerosols.

## Introduction

Aerosols are abundant in the atmosphere and play a key role in air pollution, human health and climate^1,2^. Secondary organic aerosol (SOA) often makes up the majority of submicron atmospheric aerosol particles by mass^3^. SOA mostly forms through the oxidation of volatile organic compounds (VOCs) emitted from biogenic or anthropogenic sources^4^. In the atmosphere, SOA frequently becomes internally mixed with particles composed of salts (e.g., ammonium sulfate; (NH_4_)_2_SO_4_), mineral dust or particles emitted from combustion and industrial processes^5^. These particles often contain transition metals such as iron (Fe). For example, on a local scale in urban regions, combustion and industrial emissions have been identified as major sources of particulate Fe. In these particles, dissolved i.e., available Fe, has been found to contribute up to 80% of total Fe^6^. On a global scale, mineral dust remains the largest contributor of Fe^7^. Moreover, mixing of SOA with Fe-containing particles can facilitate additional dissolution of minerals and formation of metal-organic complexes^8–10^. Thus, understanding the formation and chemistry of these complexes, in particular Fe-organic complexes is of utmost importance. They can act as a sink for particle-phase organics and can drive photochemical reactions in these aerosols, with important implications on atmospheric chemistry and public health.

Photochemical reactions initiated by Fe-organic complexes start with ligand to metal charge transfer (LMCT), resulting in the breakup of the Fe-complexes, reducing the Fe and oxidizing the ligand (L):

R1 $${\rm{Fe}}\left({\rm{III}}\right){\mbox{-}}{\rm{L}}+{hv}\to {[{\rm{Fe}}\left({\rm{III}}\right){\mbox{-}}{\rm{L}}]}^{* }$$

R2 $${\left[{\rm{Fe}}\left({\rm{III}}\right){\mbox{-}}{\rm{L}}\right]}^{* }\to {\rm{Fe}}\left({\rm{II}}\right)+{\rm{L}}\cdot$$

When the ligand is a carboxylate (COO) ion - formed when Fe complexes with carboxylic acids - decarboxylation reactions can occur, generating CO_2_ and organic radicals:

R3 $${\rm{R}}{\mbox{-}}{\rm{COO}}\cdot \to {\rm{R}}\cdot +\,{{\rm{CO}}}_{2}$$

R4 $${\rm{R}}\cdot +\,{{\rm{O}}}_{2}\to {{\rm{RO}}}_{2}\cdot$$

The generated peroxyl radicals (RO_2_) can induce the production of other reactive oxygen species (ROS), such as HO_2_ and H_2_O_2_ or organic peroxides. Therefore, this LMCT driven chemistry ‘ages’ SOA by directly altering the physicochemical properties of SOA, and presents a major sink for common SOA species, such as carboxylic acids, in the troposphere. In addition to changing the physicochemical properties of aerosol through particle phase reactions, ROS also increases particle oxidative potential^11,12^, which is thought to be key for the health impacts of the aerosols. So far, research has mostly focused on understanding this photochemistry through the use of SOA model systems, such as phenols, diacids and carbonyl Fe-complexes^13,14^.

The photochemistry of Fe complexes in aqueous systems has been well studied due to the importance of Fenton chemistry, i.e., cycling of Fe(II) and Fe(III)^8^, for wastewater processing^15–17^. In the presence of organics, the oxidation of Fe(II) can be driven by peroxides, forming ROS, like OH and RO radicals^11^:

R5 $${\rm{Fe}}\left({\rm{II}}\right)+{{\rm{H}}}_{2}{{\rm{O}}}_{2}\to {\rm{Fe}}\left({\rm{III}}\right)+\cdot {\rm{OH}}+{{\rm{OH}}}^{-}$$

R6 $${\rm{Fe}}\left({\rm{II}}\right)+{\rm{ROOH}}\to {\rm{Fe}}\left({\rm{III}}\right)+\cdot {\rm{OH}}+{{\rm{RO}}}^{-}$$

R7 $${\rm{Fe}}\left({\rm{II}}\right)+{\rm{ROOH}}\to {\rm{Fe}}\left({\rm{III}}\right)+{\rm{RO}}\cdot +\,{{\rm{OH}}}^{-}$$

R8 $${\rm{Fe}}\left({\rm{II}}\right)+{\rm{ROOR}}\to {\rm{Fe}}\left({\rm{III}}\right)+{\rm{RO}}\cdot +{{\rm{RO}}}^{-}$$

Reduction of Fe(III) to Fe(II) is primarily driven through photochemical processes initiated by UV/visible light^18^. For example, via:

R9 $${\rm{Fe}}\left({\rm{III}}\right){\mbox{-}}{\rm{OH}}+h\nu \to {\rm{Fe}}\left({\rm{II}}\right)+{\rm{HO}}\cdot$$

And the photochemical degradation of Fe-organic complexes shown in R1 to R4.

More recently, studies of Fenton chemistry have expanded to include Fe-driven reactions under dilute cloud-like conditions^8,19–24^. However, very little is known regarding this photochemistry under non-ideal, and relatively concentrated, aerosol conditions. Fe(III)-citrate has been used as a model system to study the photochemistry of atmospheric SOA^12,14,25–27^. In these studies, the oxidation of citric acid was explored, providing a proxy mechanism for atmospheric carboxylic acid removal in atmospheric aerosol. This was investigated by initiating peroxy radical chemistry, through Fe(III)-citrate photochemistry, which produces ROS via Fenton-induced production of HO_2_, OH, H_2_O_2_ and organic peroxides. The work with Fe(III)-citrate revealed the role of key parameters affecting this photochemistry, like the importance of O_2_ availability for driving this peroxy radical chemistry. It also revealed how diffusion limitations resulting from high aerosol viscosity at lower RH, can slow down photochemical processing^26,27^.

However, the chemical complexity of ambient SOA is much greater than these simple model systems. For example, Weller et al. demonstrated that the quantum yield and hence photochemical reduction of Fe, varied depending on the molecular functionality of Fe-carboxylate complexes^28^. Additionally, organic species themselves can often act as photosensitizers^29–33^ or be photolabile^34,35^, limiting our understanding of the role Fe plays in the photochemical aging of chemically complex ambient SOA.

Here, we discuss the photochemical aging of chemically complex SOA, formed in an atmospheric simulation chamber on Fe-containing (NH_4_)_2_SO_4_ seed particles. SOA was formed via dark α-pinene ozonolysis at high and low RH conditions. For each RH condition, particles were collected both pre- and post-irradiation with UV light in the atmospheric simulation chamber for offline analysis of their chemical composition and functionality, using Scanning Transmission X-ray Microscopy with Near-edge X-ray Absorption Fine Structure (STXM/NEXAFS) spectroscopy and Fourier Transform Infrared (FTIR) spectroscopy. The photochemical reduction of Fe in the different SOA samples was also evaluated in-situ, using a specialized environmental cell. This allowed particles to be irradiated in-situ while being exposed to defined RH and oxidant conditions. We discuss the impact of Fe and atmospheric conditions such as RH (and hence particle mixing state) on SOA formation and composition in the context of Fe-complex formation and the photochemistry of Fe-containing SOA.

## Results

### SOA functionality from NEXAFS carbon spectra

**Fig. 1:**
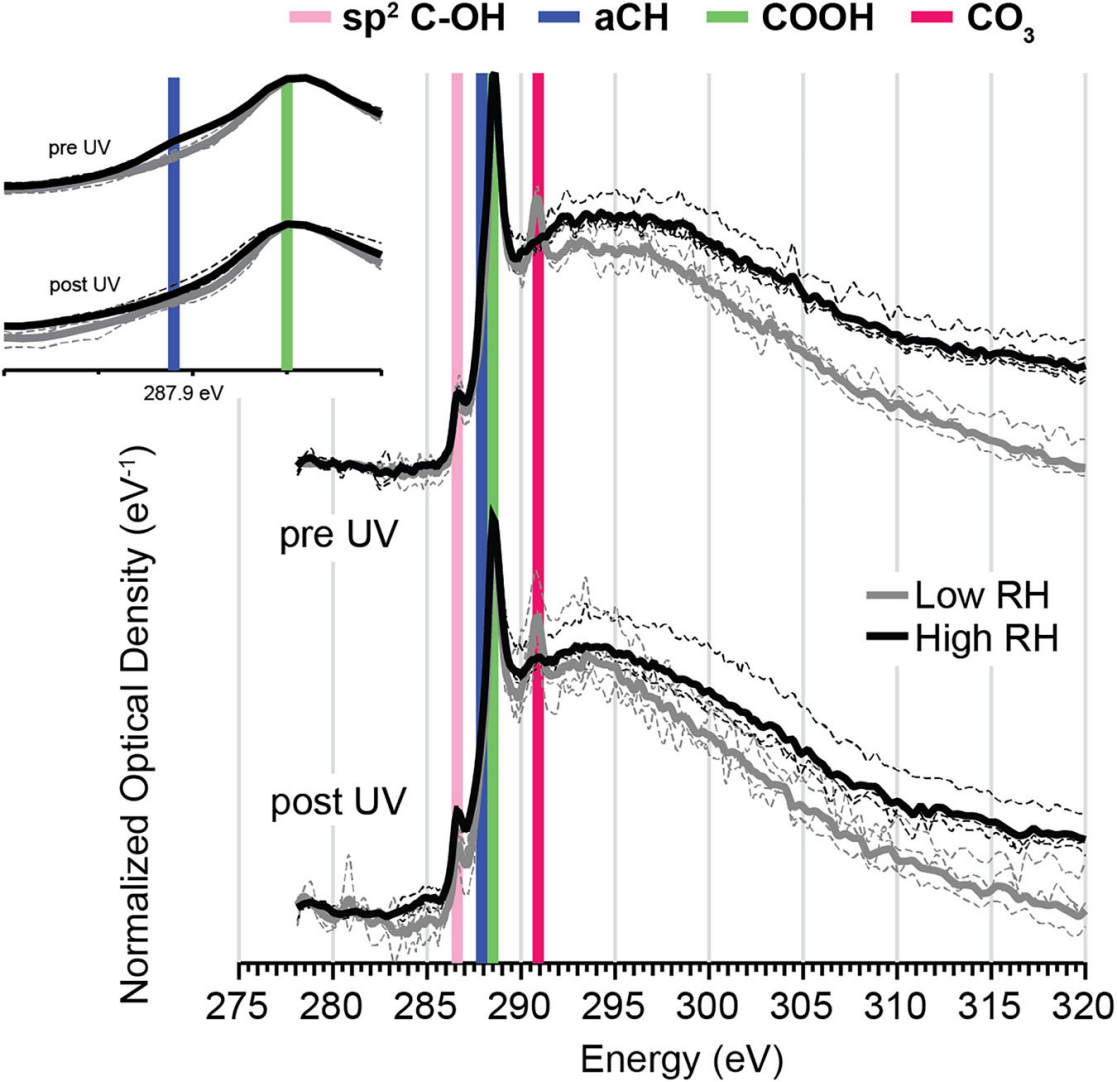
Carbon K-edge NEXAFS spectra for Fe-containing α-pinene SOA. Near edge fine structure absorption spectra at the carbon K-edge for Fe-containing SOA formed at low RH (grey traces, <10%) and high RH (black traces, >80%), and collected pre- and post-UV irradiation at 350 nm in the atmospheric simulation chamber. All spectra have been corrected for pre-edge background absorption intensities and are shown as optical densities normalize to the COOH peak at 288.5 eV (eV^−1^). Spectra are shifted vertically for clarity and better comparability. Averaged spectra for different samples are shown as the solid lines. The dashed lines represent average spectra for individual samples containing between 1 to 6 particles (summarized in Supplementary Table 1). sp^2^ C-OH = unsaturated OH group, aCH = alkane, COOH = carboxylic acid, CO_3_ = carbonate.

Averaged carbon spectra at the K-edge of Fe-containing α-pinene SOA formed at low and high RH in the atmospheric simulation chamber and measured under vacuum in the STXM/NEXAFS are shown in Fig. 1. This includes samples collected before and after photochemical aging, labeled “pre-UV” and “post-UV”, respectively. These spectra depict the functionality of the particles. All spectra showed similarities, with the presence of a large peak at 288.5 eV, which was attributed to the C 1 s → π*_R(C*=O)OH_ transition of carboxylic acid groups (COOH). For pre-UV samples, this peak was enhanced for SOA formed at low RH - when evaluating differences in peak height compared to the post-edge around 300 eV - compared to SOA formed at high RH. Thus, more COOH groups were present per total SOA carbon mass at low RH. It should be noted that esters and carboxylic acids appear in a similar energy range in Fig. 1, and hence may also contribute to the observed signal. Additionally, all samples showed a smaller peak around 286.6 eV, which is similar to the C 1 s → π*_phenolic(C*-OH)_ transition^36^. We attribute this to unsaturated (i.e., sp^2^ hybridized C) OH containing groups (sp^2^ C-OH), since phenolics themselves are not expected to be present in our samples. For SOA formed at low RH there was an additional peak at 290.9 eV, which was assigned to a C 1 s → π* _C*O3_ carbonate transition (CO_3_), previously associated with beam damage^37^. The CO_3_ peak was more predominant in spectra averaged over the center of the particle (vs. outer regions of a particle; Supplementary Fig. 1). This could indicate not only phase separation of the particles but that beam damage was somehow enhanced the presence of inorganics. Although the mechanism through which this would occur remains unclear. Lastly, the SOA formed at high RH showed a slight peak ‘shoulder’ around 287.9 eV in the pre-UV sample, which disappeared after irradiation. This peak was attributed to the C 1 s → σ*_R(C*-H)R_ transition of unsubstituted alkane groups (aCH), and suggested that when formed at high RH a loss of unsubstituted carbon occurred, perhaps due to photochemically driven fragmentation or functionalization reactions. This was not the only spectral change that was observed following photolysis. For SOA formed at both low and high RH, the COOH peak height (compared to the post-edge at 300 eV) was less prominent after photochemical aging, i.e., in post-UV samples. This is consistent with decarboxylation reactions and loss of CO_2_^14^. Decarboxylation was further evident in the spectra for the SOA formed at high RH, where the normalized optical density of the carbon post-edge (300–320 eV) was shown to decrease following photolysis. This indicates total mass loss of carbon likely from loss of CO_2_. The intensity of the CO_3_ peak was also observed to increase slightly following photolysis, for the low RH sample. This suggests that this peak resulted at least in part from degradation of organics present in the SOA, although it remains unclear whether this was due to photolysis or beam damage as mentioned previously. Our STXM/NEXAFS spectra also showed similarities to those of other monoterpene + O_3_ derived SOA (e.g., Supplementary Fig. 2 of Laskin et al.^38^), which also had an abundance of COOH and sp^2^ C-OH groups.

### Functionality of SOA as determined by FTIR

**Fig. 2:**
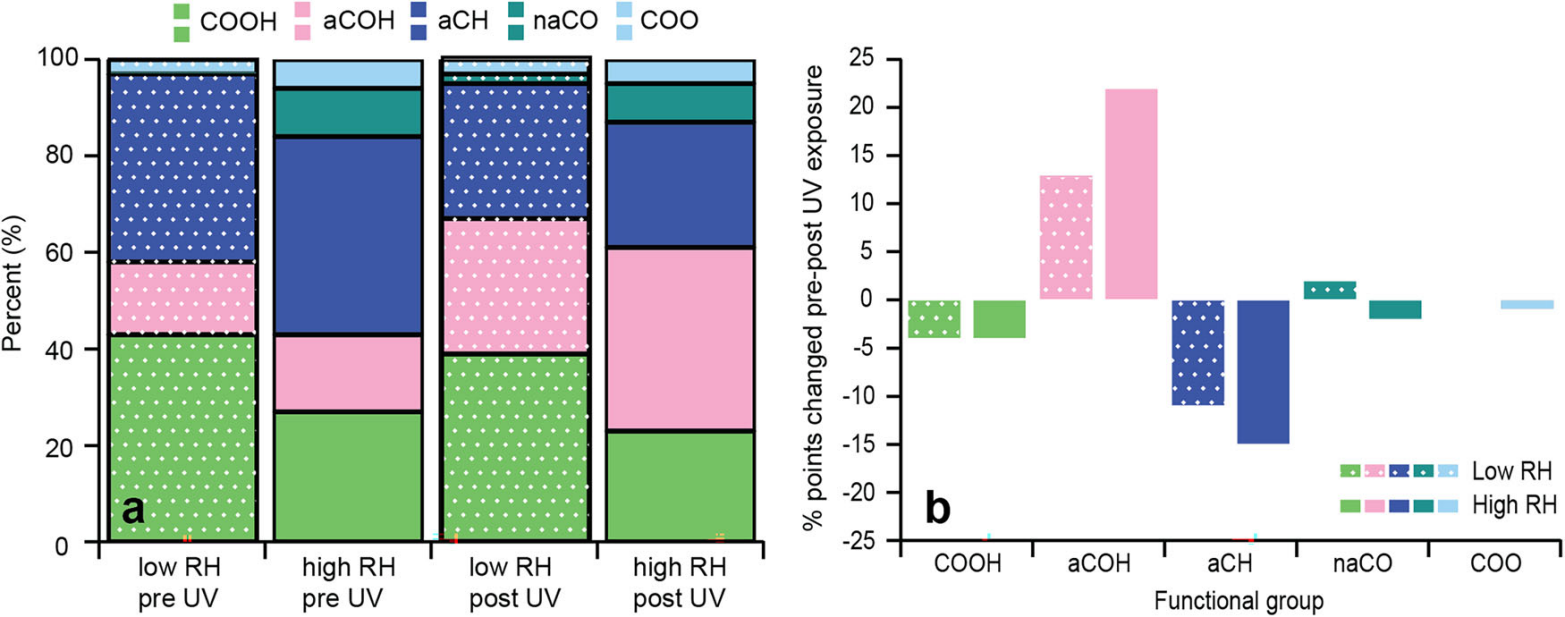
Functionality of Fe-containing *α*-pinene SOA. **a** Relative contribution of major functional groups present in SOA formed in an atmospheric simulation chamber under low RH (<10%; colours with pattern) and high RH (>80%; plain colours) conditions, and for pre- and post-UV exposure, respectively. Percentages give contribution to total organic mass as determined by FTIR. **b** Percent point change in functional group contribution following photolysis in the atmospheric simulation chamber for SOA formed under low RH and high RH conditions. A summary of the data included in this figure is shown in Supplementary Table 2, and deviation between replicate measurements in Supplementary Fig. 5. COOH = carboxylic acid, aCOH = alcohol, aCH = alkane, naCO = non-acid carbonyl, COO = carboxylate.

Analysis of samples was also conducted using FTIR, to provide additional chemical characterization of SOA. FTIR has increased sensitivity towards functional group selectivity compared to STXM/NEXAFS. Figure. 2a demonstrates that the functionality of SOA formed at both low and high RH were comprised predominantly of five main organic groups: carboxylic acids (COOH), alcohols (aCOH), alkanes (aCH), non-acid carbonyls (naCO), and carboxyl (COO). A sample spectrum showing the peak fitting applied for the various functional groups is shown in Supplementary Fig. 2 and Supplementary Note 1. The most abundant functional groups for all pre-UV samples were aCH (39% for SOA formed at low RH and 41% for SOA formed at high RH), and COOH (43% for SOA formed at low RH and 27% for SOA formed at high RH). These were also dominant in the NEXAFS spectra (Fig. 1) for all samples, and consistent with existing literature for both laboratory and ambient measurements^39,40^. Furthermore, the FTIR measurements revealed that SOA formed at low RH had more COOH functionality compared to SOA formed at high RH (43 vs. 27%, respectively) pre-UV exposure. One possible explanation for this lower relative abundance of COOH is conversion of acids to dimers (via an ester linkage^41^, noting that esters would not appear as COOH in FTIR spectra) driven by enhanced H abstraction of acids by OH formed via e.g., R6, at high RH. This interpretation agrees with the naCO functionality observed here, and abundance of dimers in SOA formed at >80% RH observed previously^42^. In addition, the SOA formed at high RH contained naCO, (e.g., from esters, ketones or keto acids), and aCOH groups, consistent with the sp^2^ C-OH peak at 286.6 eV in the NEXAFS spectra (Fig. 1). All samples also had a peak in the FTIR spectra around ~1650 cm^−1^, which was attributed to a carbon-oxygen resonance from the carboxylate group (COO)^43^. This carbon-oxygen resonance has a distinct infrared signature from COOH, which is characterized by broad O-H absorption in the region between 2500 and 3200 cm^−1^, and other carbonyls which have carbon-oxygen stretches at frequencies typically greater than 1700 cm^−1^. Furthermore, this COO feature has been reported in literature for Fe-carboxylate systems (see Supplementary Note 2)^43^. Interestingly, this COO peak was more abundant in SOA formed under high RH conditions (6%) in comparison to low RH (3%). This is perhaps from an enhancement in Fe-carboxylate complexes formed at high RH (observed here as COO), which might also help explain why fewer COOH groups were observed.

As with the NEXAFS spectra, the relative contribution of different functional groups changed following photolysis for all samples (Fig. 2b). Most notably, a decrease in both COOH and aCH groups occurred. At the same time an increase in aCOH and naCO (in the case of SOA formed at low RH) was observed. As mentioned previously, decarboxylation following photolysis is well known, e.g.^14^, and could help rationalize loss of COOH functionality in post-UV samples. Interestingly, more than a 10% decrease in aCH functionality (i.e., unsubstituted carbon) was observed for both SOA formed at low and high RH. This may be due to direct photolysis and fragmentation of monomers or dimers followed by evaporation of more volatile products^35^. Alternatively, direct or indirect fragmentation of species formed through photo-Fenton reactions (e.g., RO radicals, OH radicals, etc.) could contribute to loss of aCH. Likewise, SOA formed at high RH exhibited a larger increase in aCOH (22%) post-UV compared to SOA formed under low RH (13%). This increased aCOH functionality may have been enhanced at high RH due to photo-Fenton chemistry; for example, from OH addition to R and RO radicals, which form directly or indirectly (e.g., degradation of RO_2_ to RO) via photo-Fenton reactions. Quantifying ROS through methods such as spin-trapping or florescence assays would help clarify this mechanism. Furthermore, a decrease in the COO peak was observed post-UV for high RH samples - perhaps from break up of photochemically active Fe-carboxylates - whereas no change in this peak was noted for SOA formed at low RH. Overall, these results suggest that SOA from dark ozonolysis of α-pinene formed under low and high RH conditions in the presence of Fe-containing inorganic seeds not only has different initial chemical composition and functionality but is also impacted by photolysis in distinct ways.

### Photochemical reduction and re-oxidation of Fe-containing α-pinene SOA particles

To explore the photochemical changes in detail, particles were collected on silicon nitride (SiNit) membranes directly from the atmospheric simulation chamber (i.e., pre-UV). The SiNit membranes with the particles where then mounted in a specialized environmental cell that can be coupled for STXM/NEXAFS measurements. The environmental cell allowed us to expose the particles to 375 nm UV light (to reduce Fe(III) to Fe(II)), and ~300 ppb O_3_ (to re-oxidize the Fe(II) to Fe(III) following the UV-induced reduction) at defined RH, while in-situ monitoring photochemical reactions using STXM. Particle collection and analysis are described in detail in the Methods section. These conditions were chosen to be similar to the irradiation and oxidation conditions in our atmospheric simulation chamber experiments.

**Fig. 3:**
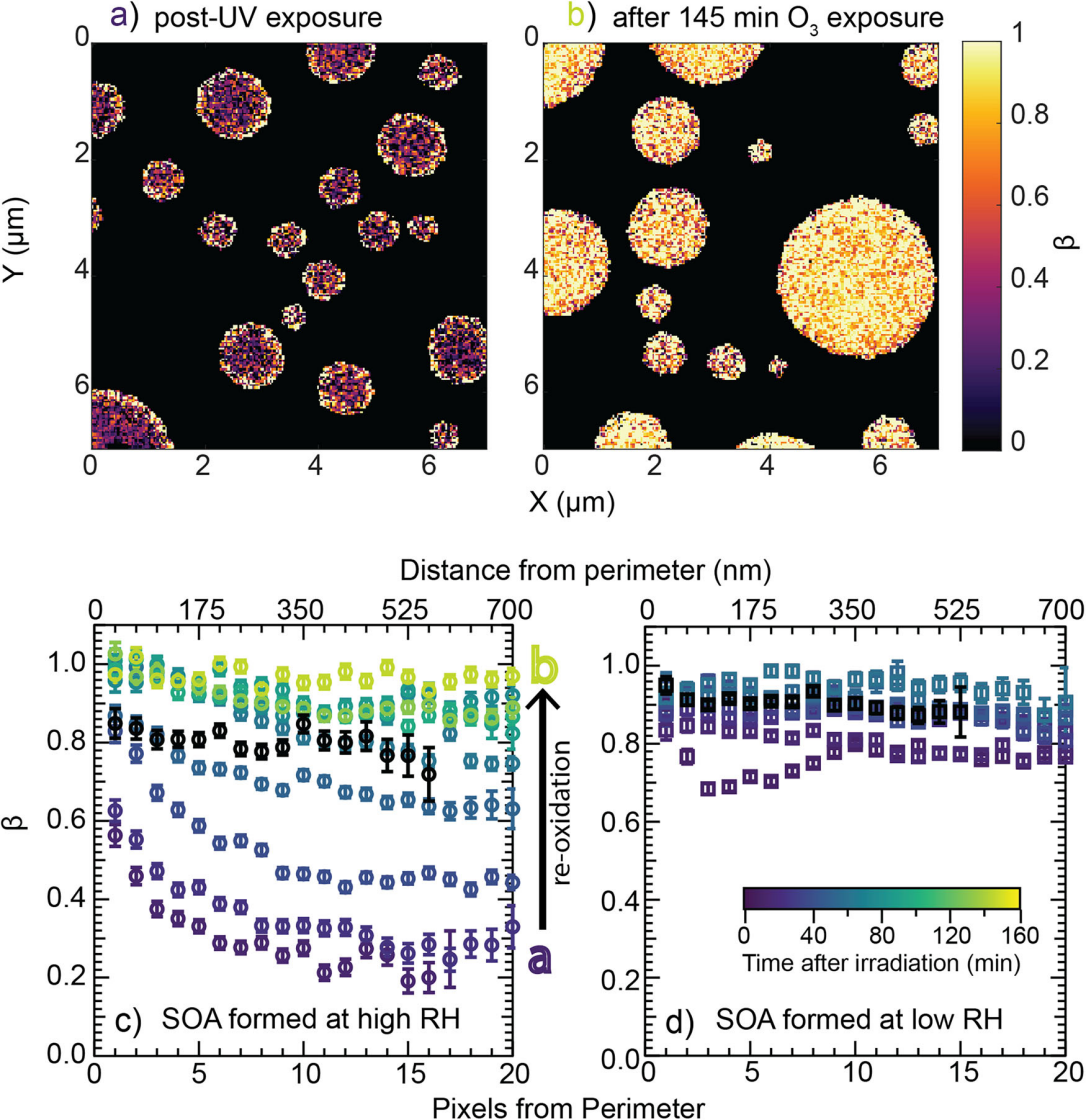
Photochemical reduction and re-oxidation of Fe-containing SOA. Chemical ‘maps’ of particles showing the spatially-resolved average Fe(III) fraction, β ([Fe(III)]/[Fe(II)+Fe(III)]), for particles that were collected from the atmospheric simulation chamber at high RH (a/b/c) and low RH (d) and put in the STXM environmental cell at ~80% RH (all panels). (a/b) β across particles formed at high RH, where **a** shows particles post-UV exposure (at ~375 nm) within the environmental cell (i.e., after UV reduction) and **b** shows particles 145 min after the addition of ~300 ppb O_3_ to the environmental cell, (i.e., after re-oxidation by O_3_). A summary of the particle maps during this re-oxidation in the presence of ~300 ppb O_3_ (time between **a** and **b**) for the SOA formed at high RH is shown in (**c**), where β is shown averaged from the pixels from the particle perimeter, i.e., as a proxy distance from particle edge. The data points are shown for individual time points (same colour). Exemplary error bars are 1 σ standard deviation (**d**). The summary of particle maps for Fe in SOA formed at low RH (<10%) in the atmospheric simulation chamber but irradiated in the STXM environmental cell at ~80% RH in the presence of ~300 ppb O_3_. The black data points in (**c**) and (**d**) show the average β before irradiation. A summary of the data shown in Fig. 3c/d is listed in Supplementary Tables 3 and 4.

Figure 3 shows ‘maps’ of the spatially resolved fraction of Fe(III) of total Fe within the particles, i.e., [Fe(III)]/[Fe(II)+Fe(III)], termed β. The substrate is shown in black and the SOA particles correspond to the dark violet to yellow coloured pixels, each corresponding to a 35 × 35 nm area. The maps in Fig. 3a depict SOA particles formed at high RH in the atmospheric simulation chamber, which were exposed to the same high RH (~80%) in the STXM environmental cell, where they also underwent UV irradiation for 9 min. The dark violet colours denote generally lower β values (low Fe(III) fraction), as would be expected following photochemical reduction of Fe, i.e., conversion of Fe(III) to Fe(II) as shown in R1 to R3. Continuous mapping was then conducted in the dark while particles were exposed to O_3_ (to reoxidize Fe(II) to Fe(III)). Subsequent maps were conducted across different particles of the same sample, i.e., each new map targeted a neighbouring region of particles to the previous map, to minimize the impact of beam damage (Supplementary Note 3). In the presence of O_3_, β gradually increased to 1.0 over time (Fig. 3b), indicating full re-oxidation of Fe. A summary of the temporal evolution of β observed during these experiments/mapping is shown in Fig. 3c. It depicts the average β as a function of the pixels from perimeter, i.e., distance from particle edge, where the edge corresponds to 0 and one pixel is equal to 35 nm. The coloured symbols in Fig. 3c/d correspond to different times after the UV irradiation in the environmental cell was stopped. The presence of a gradient in β, high at the perimeter and low in the particle center (bulk) during the initial phase of reoxidation (<60 min) suggests reacto-diffusive limitation; i.e., the oxidants (O_3_ and O_2_) are getting depleted towards the particle interior and Fe(II) is not diffusing fast enough outwards to get oxidized. The average β over the duration of the re-oxidation mapping was used to calculate a re-oxidation rate of Fe(II) (Supplementary Fig. 8), which was determined to be (4.6 ± 0.6) × 10^-4 ^s^-1^ for the data shown in Fig. 3c. This is at least an order of magnitude slower than the photoreduction of Fe(III). In the presence of lower O_3_ concentrations (~ 20–30 ppb) the rate of Fe re-oxidation was ~4 times slower ((1.2 ± 0.5) × 10^−4 ^s^−1^; Supplementary Fig. 9). By contrast, when SOA formed at low (<10%) RH in the atmospheric simulation chamber was exposed to similar conditions in the STXM environmental cell - i.e., irradiated at ~80% RH when particle inorganic and organic fractions would be more homogeneously mixed (see Supplementary Note 4), and exposed to ~300 ppb O_3_ - we did not observe significant reduction of Fe(III) (Fig. 3d). This lack of Fe(III) reduction was also observed when SOA formed in the atmospheric simulation chamber at low RH were irradiated in the STXM environmental cell at 0% RH (Supplementary Fig. 11). We point out that our previous experiments using Fe-citrate demonstrated photochemical reduction of Fe under all conditions tested in the environmental cell, i.e., regardless of the RH or presence/absence of an oxidant in the cell^14^.

In summary, Fe(III) in SOA formed at high RH, was photochemically reduced to Fe(II) when exposed to UV light and subsequently re-oxidized to Fe(III) in the presence of O_3_. In contrast, Fe(III) in SOA formed at low RH, did not reduce to Fe(II) under UV light, even when exposed to high RH conditions in the STXM environmental cell. This suggests that chemical differences in the SOA and not just microphysical processes, e.g., diffusion limitations or mixing of Fe and SOA, impact the photochemistry of Fe-containing SOA.

## Discussion

Figures 1–3 provide clear evidence that the RH conditions during SOA formation (in the presence of Fe-containing seed particles), impacts the composition/functionality of condensed-phase organics and formation of Fe-complexes. Furthermore, the impact on Fe-complex formation is highlighted by the differences observed in the photochemical reduction of Fe in SOA formed at high vs. low RH shown in Fig. 3c, d.

Fe is known to complex with organic and inorganic compounds^8^, and we expect both to form in our internally mixed inorganic-organic aerosols. Ambient measurements have shown that Fe-carboxylates often form when SOA mixes with Fe-containing particles^44^. Furthermore, these complexes can undergo photochemistry at around ~350–375 nm (Supplementary Table 5), which was the wavelength range used in our experiments. The presence of photochemically active Fe-carboxylate complexes in SOA formed at high RH is supported by the reduction of Fe (Fig. 3c) and the abundance of functional groups characteristic of carboxylates (Figs. 1 and 2). These include COOH groups measured by STXM/NEXAFS and FTIR, as well as the carbon – oxygen resonance (from COO) in the FTIR spectra at ~1650 cm^−1^. Additionally, the decrease in the relative abundance of this COO peak following photolysis for SOA formed at high RH - likely from the breakup of Fe-carboxylate complexes following photolysis - provides further evidence of their presence in our aerosol.

The abundance of other functional groups such as COH and naCO observed by FTIR - and known multifunctionality of α-pinene oxidation products^45^ - suggest the Fe-carboxylates present in our aerosol likely also contain additional functional groups. This additional functionalization of carboxylates has been shown to enhance photochemical reduction of Fe-carboxylates, e.g., through conversion of an α-COH to a ketone or aldehyde during cleavage of COOH^46^. Unfortunately, STXM/NEXAFS and FTIR only allow us to determine bulk functionalization and not that for individual molecules. The complexity of SOA and limited literature for the structure and functionality for many condensed phase species further complicate exact identification of carboxylates present in our particles. However, these multifunctional Fe-carboxylate likely include species such as keto/diol containing carboxylates, since for example, keto acids are known terpene oxidation products^45^. Interestingly, in previous atmospheric simulation chamber experiments using online mass spectrometry^42^, we observed dark reduction of Fe. Similarly, Weller et al.^28^ reported Fe reduction under dark conditions in the presence of keto/diol containing Fe-carboxylates. The presence of keto/diol containing Fe-carboxylates might also help rationalize why the β for samples where SOA was formed at high RH, was lower pre-UV than for samples formed at low RH (β = ~0.9 vs. ~0.8, respectively; Fig. 3c/d), indicating a higher initial Fe(II) fraction.

In comparison to SOA formed at high RH, where Fe(III) was fully reduced to Fe(II) (Fig. 3c), there was minimal reduction of Fe in SOA formed at low RH but photolyzed at high RH (Fig. 3d). From this it can be concluded that the same photochemically active Fe-carboxylate complexes are either not formed, or formed to a lesser degree at low RH. At low RH (<10%) we expect limited mixing between the inorganic seed particles and SOA that condense and partition onto them, restricting the formation of Fe-carboxylate complexes. Contrarily, at high RH (>80%) seed particles and SOA should rapidly mix, allowing for the formation of more Fe-organic complexes, including photochemically active Fe-carboxylates, during initial SOA growth. This is supported by data shown in Fig. 3, and mass spectrometry data from our previous work; we observed rapid disappearance of the Fe(II) signal following the onset of SOA formation at high RH (Supplementary Fig. 12), which we attributed to quick mixing and oxidation of Fe(II) in seed particles by organics in SOA. In other words, with mixing limitations at low RH we could expect favoured formation of Fe-inorganic complexes. Furthermore, when SOA formed at low RH were exposed to high RH conditions in the STXM environmental cell, there was no evidence for the formation of photochemically active complexes (i.e., Fig. 3d) upon mixing of inorganic and organic aerosol fractions.

It is well established that in aqueous systems Fe can complex with hydroxides and water, in pH-dependent equilibria^8^. The pH of our bulk Fe/AS solution was measured and found to be around ~4.5. At this pH range, the dominant Fe hydroxy complex is Fe(OH)^2+^ ^47^. Since our seed particles are generated from a mixture of FeSO_4_ and (NH_4_)_2_SO_4_ it is likely that Fe-inorganic complexes also exist, including e.g., Fe(SO_4_)^+^ ^48^. At high RH - when Fe-inorganic complexes would also be expected to form - competitive reactions of SOA with not only Fe but also e.g., SO_4_^2−^ ^49^, may limit the formation of Fe-inorganic complexes. Additionally, Fe-inorganic complexes such as Fe(OH)^2+^ and Fe(SO_4_)^+^ have a comparatively lower molar absorptivity (ε) at ~360 nm than many Fe-carboxylates (Supplementary Table 5). This lower ε of Fe-inorganic complexes at ~360 nm, would help rationalize why reduction was not observed in Fig. 3d if Fe-inorganic complexes dominated in SOA formed under low RH conditions. It should be noted that the wavelength range of our experiments was narrower than the actinic spectrum. As such, photolysis of Fe from a variety of complexes, e.g., Fe(OH)^2+^, should occur in the atmosphere more efficiently at lower wavelengths^47^. This also includes other Fe-carboxylates, which may exhibit higher quantum yields at wavelengths <350 nm. For example, unsubstituted carboxylates such as oxalates exhibit higher quantum yields at wavelengths <350 nm but lower quantum yields at ~360 nm - especially compared to other substituted carboxylates as shown in Supplementary Table 5.

One thing that remains unclear is that despite the presence of a peak for COO in the SOA formed at low RH, the Fe did not undergo photochemical reduction (Fig. 3d). As mentioned above, it is possible the Fe-carboxylates formed at low RH may simply exhibit lower quantum yields at the wavelength range studied here, than the Fe-carboxylates formed at high RH. This could be driven through compositional differences in the carboxylates formed (e.g., additional functionality) or the metal-to-ligand ratio, which have been shown to impact the photochemical reduction of Fe-carboxylate complexes^28,43^. For example, Fe-oxalates with a 1:2 metal-to-ligand ratio have a generally higher quantum yield than complexes with a 1:3 metal-to-ligand ratio^50^. This difference could also be attributed to both homo- and mixed- ligand complexes, the latter of which are thought to form more stable complexes than the former^51^. It is also possible that other non-carboxylate Fe-complexes form, which are also photochemically inactive in the wavelength range studied here. However, this would require further studies using a more targeted approach, e.g., via chromatographic analysis and photolysis experiments using model systems, to both identify and determine the formation and photochemical activity of these complexes. Understanding these factors is critical to systematically evaluate the impact of this chemistry across the broader actinic spectrum, where there is significant solar irradiance at wavelengths <350 nm. Additionally, SOA formed at low RH still exhibited changes in functionality despite a lack of photochemical reduction in Fe. As such, understanding the impact of aerosol mixing state on other aging processes, such as ‘bleaching'^13^ or other photochemical processes^35,52,53^ in internally mixed aerosol systems containing transition metals is important.

Here we explored the photochemistry of SOA, formed from dark ozonolysis on Fe-containing (NH_4_)_2_SO_4_ seeds particles, under low and high RH conditions. We gained novel insights into the important interplay between atmospheric conditions and SOA physicochemical properties on the formation of Fe-containing complexes and their role in photochemical aging of aerosol particles. Although we were not able to identify the exact chemical composition of the ligands in the Fe complexes in our particles, our results provide clear evidence that when chemically complex SOA is formed and becomes mixed with Fe-containing seed particles under different RH conditions, different Fe-complexes form. These Fe-complexes likely comprise both organic and inorganic complexes, including Fe-carboxylates and Fe-hydroxides. This has important implications on sinks for species such as carboxylic acids, which will form photochemically active Fe-carboxylate complexes when SOA and Fe-containing particles become mixed at higher RH, and hence atmospheric chemistry/composition overall. Furthermore, our results demonstrate that SOA formation conditions such as RH can regulate the photochemistry of aerosol, even after initial SOA formation. This was evidenced when Fe present in SOA formed at low RH did not undergo photochemical reduction, even when exposed to high RH conditions in our STXM environmental cell, i.e., when particle inorganic and organic fractions would have been more completely mixed. This highlights that chemical composition alone, cannot be used as a predictor for Fe-complex formation, especially when Fe becomes mixed with chemically complex SOA. To understand the true impact of Fe-complex driven photochemistry on SOA aging - and its broader implications on climate and air quality - atmospheric conditions such as RH during formation clearly need to be taken into account as well.

## Methods

### SOA generation

Samples were generated in an ~8 m^3^ atmospheric simulation chamber at the Paul Scherrer Institute in Switzerland. The chamber and generation of the SOA have been described previously^42^. Briefly, α-pinene SOA were generated via dark ozonolysis in the presence of Fe-containing seed particles. Experiments were conducted under both low (<10%) and high (>80%) RH conditions, to explore the impact of aerosol mixing. Seed particles were generated using a ~1000 ppb solution containing a mixture of ammonium sulfate ((NH_4_)_2_SO_4_) and FeSO_4_, which had a 50:50 mol ratio of NH_4_^+^: Fe(II). Humidified seed particles were nebulized into the chamber until an aerosol mass density of ~70 μg m^-3^ was obtained as measured by a scanning mobility particle sizer (SMPS). SOA was formed and grown onto seed particles by adding ~300 ppb O_3_, followed by ~50 ppb α-pinene, to the atmospheric simulation chamber in the absence of light. A mass concentration of ~60 μg m^-3^ SOA formed for experiments at low RH, whereas ~120 μg m^-3^ formed under high RH conditions. This resulted in a ~1:1 mass ratio of seed : SOA for the low RH conditions and ~2:1 ratio for high RH conditions.

### Aerosol particle collection

Aerosol particle samples were collected both before (pre-) and after (post-) photolysis to explore changes in chemical composition and morphology due to irradiation with UV light. Pre-photolysis samples were collected ~1 h after the addition of α-pinene into the chamber when SOA mass had reached a maximum and gas-phase α-pinene had been fully consumed. Post-photolysis samples were collected after ~30 min of irradiation, which started ~3 h after α-pinene was injected into the chamber and ~2 h after aerosol had reached a maximum mass (end of SOA formation).

Aerosol were collected using a single jet impactor, which has been describe previously^26,27^. A summary of samples collected is shown in Supplementary Fig. 13 and Supplementary Table 6. Briefly, aerosol particle samples were impacted onto either Cu TEM grids (Carbon Type-B 400 mesh Cu, Ted Pella Inc.) or silicon nitride (SiNit) membranes (50 nm thick, 0.5 × 0.5 mm, Silson Ltd.), at a flow of ~0.7 lpm for 1.5 min. The 2.5 mm Cu TEM grids were used for analysis under vacuum, whereas the SiNit membranes for analysis in the STXM environmental cell (described below). Prior to collection the SiNit membranes were sealed onto mounting clips using an adhesive wax (Crystalbond 509 mounting adhesive, SPI supplies). To minimize further oxidation and photochemistry, samples were placed into thermally sealed bags with an oxygen scrubber (ATCO) with [O_2_] < 0.01% and desiccant (RS PRO) with RH < 10% and stored at room temperature until analysis.

Immediately prior to analysis, the Cu TEM grids were mounted in the dark onto a sample card and then stored briefly in the dark under vacuum. For the STXM environmental cell experiments, all mounting of samples in the vacuum chamber was done in the dark to minimize potential photochemistry.

### Chemical characterization by STXM/NEXAFS

Single particle chemical characterization was achieved by STXM/NEXAFS using the PolLux facility at the Swiss Light Source, which is located at the Paul Scherrer Institute, Switzerland. STXM/NEXAFS have been described in detail previously^54–56^. Briefly, monochromatic X-rays are focused to a fixed point on a sample. By scanning the sample with sub nanometer precision and measuring the transmitted photons through the particles present at different locations, an X-ray optical density (OD) image is generated. Where OD, can be determined using the Beer-Lambert’s law:

1 $${OD}=-{\mathrm{ln}}\left[\frac{I}{{I}_{0}}\right]=\rho \mu d=\sigma d,$$

and *I* and *I*_*0*_ are the transmitted and incident photon flux as a function of energy, *ρ* is the sample density, μ is the mass absorption coefficient, *d* is the sample thickness and *σ* is the mass absorption cross section. The uncertainties for *I* and *I*_*0*_ are σ_I_ = √*I* and σ_I0_ = √ *I*_*0*_ respectively. OD images were acquired at multiple discrete energies (E), i.e., a stack, which was aligned and processed using publicly available software. Since STXM/NEXAFS is a bulk sensitive technique, particles must be sufficiently small (~ 0.1–2 μm in diameter) so that X-ray transmission through the sample is possible, where reduction in signal intensity occurs via both absorption and electron excitation in atoms.

Soft X-rays (λ = 1–10 nm) are used to initiate electronic transitions, such as 1 s → π* and 1 s → σ*, which result in the observed absorption peaks. The π* excitations result from transitions between relatively low energies into orbitals of the lowest unoccupied molecular orbitals (conductive band), whereas σ* represents transitions occurring at higher energies, i.e., excitation of core electrons to energies about the Fermi level (ionization threshold). As such, X-ray absorption is sensitive to the chemical bonding environment, resulting in spectra with characteristic features caused by the optical dipole selection rules of the absorbing atom. Additionally, multiple electronic transitions can occur at an energy, and as such the absorption measured represents the sum of these at a given energy. The energy resolution, d*E*, and X-ray spot size are closely correlated, and also depend on the X-ray energy and other adjustable beamline settings, such as slit widths, that define the X-ray beam upstream of the sample inside the Pollux endstation. We chose d*E* here to balance our ability to distinguish closely spaced peaks and represent the Gaussian X-ray spot size with an appropriate square pixel size to derive C K-edge spectra and concentration profiles of Fe(II) and Fe(III) at the Fe L-edges. Note, the spot size is quantified as the full-width-half-max beam intensity profile. At the C K-edge, main absorption peaks were always separated by a minimum of 0.8 eV as determined from a few STXM/NEXFAS spectra measured with energy resolution of d*E* = 0.2 eV, where d*E* is defined as the ability to clearly measure two peaks with their full-width-half-max *OD* of 0.2 eV also separated by 0.2 eV. Therefore, we used d*E* = 0.7 eV in this study for C K-edge spectra, with the benefit that a larger d*E* coincides with greater photon flux and reduced scan time overall, with a pixel size of 60 × 60 nm^2^ to account for a spot size of 67 nm in diameter. At the Fe L-edges, two peaks for discriminating Fe(II) and Fe(III) are separated by 1.8 eV (Supplementary Fig. 14)^57^. We used d*E* = 0.7 and pixel size of 35 × 35 nm for a spot size of 48 nm. Fe(II) and Fe(III) were quantified from measuring the peak optical density, OD, at the peak maxima for the respective Fe-edges (see Supplementary Note 5). Their peak height ratio (i.e., used to determine β) was determined utilizing a linear combination of the peaks as parameterized by Moffet et al.^57^.

For the experiments described here, NEXAFS spectra at the C K-edge (278–320 eV) and Fe L-edge (700–735 eV) were collected. The C K-edge spectra were used to identify carbon functionalization of our generated SOA samples, whereas Fe L-edge data were used to identify Fe location within the particles and its oxidation state (Supplementary Fig. 10 and Supplementary Note 4). At the beginning of the experiment, energy offset calibrations were performed using polystyrene latex spheres for the C K-edge, and by intentional beam damage experiments which reduced Fe(III) to Fe(II) for the Fe L-edges, followed by comparison to literature values for iron chloride and sulfate standards. Additionally, a background subtraction (mean absorption between 278–282 eV for the C K-edge) was applied and C spectra normalized to the maximum OD of the COOH peak at 288.5 eV^58^, to facilitate comparison between different particles and samples. Beam damage was assessed and avoided at both the C K-edge and Fe L-edges as shown in Supplentary Note 3.

### Chemical Characterization by FTIR

FTIR samples were collected on Polytetrafluoroethylene filters (PTFE, 47 mm diameter, 1 μm pore size, Pall Life Sciences) at a flow rate of ~20 lpm for ~15 min. A 1 cm diameter PTFE spacer was used to concentrate sample and reduce total sampling time needed. The total aerosol mass loading for the FTIR samples was ~18–30 μg for the low and high RH experiments, respectively. Samples were placed in thermally sealed bags with a desiccant/O_2_ scrubber, and frozen before shipping. Thawed filters were analyzed in transmission mode with the FTIR spectrometer (Vertex 80, Bruker Optics). The average spectrum of three blank filters was subtracted from each spectrum to remove the Teflon interference, and the resulting spectra were processed in AIRSpec (an open-source software built with R and Shiny) for baseline correction and peak fitting. AIRSpec uses the peak areas to estimate the molar abundances of bonds, allocates the bonds to functional groups (FG) and finally estimates atomic abundances from FGs (to retrieve OM, OM/OC and O/C)^59^ according to the Beer–Lambert law (variations of FG abundance are linearly dependent to the absorbance).

### Photochemical experiments using the STXM environmental cell

In situ photochemical experiments were conducted using the STXM environmental cell at the Paul Scherrer Institute, which has been described previously^26,60^. Briefly, samples were illuminated in situ using a UV-LED fiber optic coupled to the STXM chamber. The sample was uniformly illuminated with a power density of 3.6 ± 0.6 W nm^−2^ in the wavelength range of 364 – 370 nm, using a collimator. This equated to a photon flux of 2.5 × 10^15^ photons cm^−2^ s^−1^. Samples were illuminated for 9 min with the UV LED, before the light was switched off and β, defined as [Fe(III)]/[Fe(II)+Fe(III)], was mapped continuously over multiple particles. During this time, particles were exposed to constant RH (either 0% or 80%) and O_2_/O_3_ concentrations ( ~ 20% O_2_ or ~300 ppb O_3_ based on an environmental cell pressure of 150 mbar), unless specified. The irradiation times, RH and O_2_/O_3_ concentrations were chosen to mimic conditions in our atmospheric simulation chamber.

## Supplementary information

Supplementary Information

## Data Availability

The data that support the findings of this study are publicly available on Zenodo at 10.5281/zenodo.15648924 and 10.5281/zenodo.15673140.
