# Supplementary material for: Photochemistry of iron-containing secondary organic aerosol is impacted by relative humidity during formation
Source: NPJ Clim Atmos Sci. 2025 Jul 1;8(1):246. doi: 10.1038/s41612-025-01109-6 (PMC12213612; doi:10.1038/s41612-025-01109-6)
Supplement: Supplementary file 1 — Supplementary Information [file 41612_2025_1109_MOESM1_ESM.pdf]

SUPPLEMENTARY INFORMATION

Photochemistry of iron-containing secondary organic aerosol is impacted by relative humidity during formation

Natasha M. Garner[1], Fabian Mahrt[1,2], Jens Top[1,3], Virginia Tadei[4,5], Kevin Kilchhofer[1,3,6] Satoshi Takahama[4,5], Imad El Haddad[1], David M. Bell[1], Markus Ammann[1]* and Peter A. Alpert[1,7]*

1: PSI Center for Energy and Environmental Sciences, 5232 Villigen, Switzerland

2: Now at: Department of Chemistry, Aarhus University, 8000 Aarhus, Denmark

3: Department of Environmental System Science, Institute for Atmospheric and Climate Science, ETH Zurich, 8092 Zurich, Switzerland

4: Laboratory of Atmospheric Processes and their Impacts, School of Architecture, Civil & Environmental Engineering, Ecole Polytechnique Fédérale de Lausanne, 1015 Lausanne, Switzerland

5: Laboratory of Environmental Spectrochemistry, School of Architecture, Civil & Environmental Engineering, Ecole Polytechnique Fédérale de Lausanne, 1015 Lausanne, Switzerland

6: Now at: Physikalisch-Meteorologisches Observatorium Davos, World Radiation Center (PMOD/WRC), Davos Dorf, Switzerland

7: Now at: XRnanotech AG, 5234 Villigen, Switzerland

email: natasha.garner@psi.ch or markus.ammann@psi.ch

This file contains:

Supplementary Notes 1 to 5

Supplementary Figures 1 to 14

Supplementary Tables 1 to 6

Supplementary References

Supplementary Note 1. FTIR spectra

The baseline correction package of AIRSpec[1] applies smoothing splines to adjust drift in aerosol spectra, accounting for substrate interferences, changes in detector response, and environmental factors. AIRSpec calculates the baseline-corrected spectra using the EDF parameter (effective degrees of freedom) that minimizes the median negative absorbance fraction (NAF), ensuring a physically meaningful spectrum. To quantify organic functional groups (FG) from FTIR spectra, AIRSpec fits Gaussian line shapes to alcohols (aCOH), carboxylic acids (COOH), alkanes (aCH), carbonyl (CO), carboxyl (COO), and ammonium (ammNH) in each spectrum, e.g., Supplementary Fig. 2. Molar abundances of bonds are estimated from peak areas, functional group abundances are estimated from bond abundances, and atomic abundances are estimated from functional group abundances. Detection limits are defined as the smallest area that can be statistically distinguished from background variations due to noise and baseline correction artifacts.[2] The total carbonyl (tCO) retrieved with AIRSpec can include contributions from carboxyl, ketone, ester, and aldehyde carbonyl without distinction in environmental samples due to the closeness of absorption bands.[3, 4] The total carbonyl (tCO) is apportioned to acid (COOH) and non-acid CO (naCO) and the constituent atomic molar abundance calculated following the approach described by Reggente et al.[1]

Supplementary Note 2. Evidence of photochemically active Fe-carboxyl complexes in FTIR spectra

FTIR spectra collected for Fe-containing SOA formed at high and low RH, both pre- and post-UV exposure, are shown in Supplementary Figs. 3 and 4, respectively. In the carbonyl absorbance region (1545−1900 cm^{-1}), peaks above 1700 cm^{-1} were attributed to carboxylic acids, while those below 1700 cm^{-1} were assigned to carboxylates.[5] Around ~1710 cm^{-1} a

peak assigned to a carbonyl stretch was observed. This peak was observed to be shifted to lower wavenumbers (~1600 – 1650 cm^{-1}) for SOA generated at high RH compared to SOA formed at low RH. This may indicate the presence of Fe-carboxylate complexes, which have been identified as a band in the FTIR spectra around ~1650 cm^{-1}.[6] This phenomenon is particularly evident in the pre-UV case, in Supplementary Fig. 3. Similarly, under ammonia exposure experiments,[5] for RH levels above 25%, the spectral region from 1500−1900 cm^{-1} showed a decreasing trend in carboxylic acids and an increasing trend in carboxylates. Together with increased ammonium absorbance, those results were attributed to neutralization of carboxylic acids by ammonia uptake. A shift of the carbonyl group from solid phase (1723 cm^{-1}) to aqueous phase (1660 cm^{-1}) was observed in oxalic acid,[5] highlighting the influence of high RH.

In the post-UV case (Supplementary Fig. 4), the loss of the carbonyl peak (~1710 cm^{-1}) is more pronounced. However, the carboxylate peak around ~1650 cm^{-1} in the high RH condition does not appear significantly stronger than in the low RH case. This observation could be due to gas/particle partitioning of the acid, which may be more volatile than metal-ligand complexes. Furthermore, a bigger difference in the spectra post and pre-UV exposure was noted for SOA formed at high RH. This difference was particularly prominent in the spectral region associated with carboxylic acid and carbonyl stretches and likely driven by the loss of COOH functionality by decarboxylation reactions.[7]

Organic functional groups in SOA, including esters, anhydrides, and carbonyls, can undergo hydrolysis upon exposure to water vapour. This process breaks down larger molecules into smaller, more volatile compounds that may evaporate or undergo further oxidation in the atmosphere. Additionally, hydrolysis alters the optical and hygroscopic properties of SOA, influencing aerosol interactions with sunlight and cloud formation.

Supplementary Note 3. Assessment of X-ray beam damage

The impact of beam damage on the sample caused by exposure to X-ray light was evaluated. Exposure of already oxidized particles (e.g., those containing Fe(III)) can lead to photochemical reaction resulting in reduction of species such as Fe(III) back to Fe(II) or in the case of carbon stacks, the loss of functional groups such as carbonyls. As such we performed repeated scans on particles and observed the changes in the NEXAFS spectra for both C K-edge and Fe L-edges.

Despite repeated exposure to X-ray radiation, the C spectra (Supplementary Fig. 6; between 277 – 320 eV) remained relatively unchanged between the 3 consecutive scans, in particular in between the first and second stack. A slight increase in the peak height (relative to peak base) at 285 eV and decrease (~10% per irradiation) in the peak at 289.X eV following repeated X-ray exposure was observed suggesting slight loss of COOH functionality and increase in C=C. In the case of Fe we observed similar changes as shown in Supplementary Fig. 7. The Fe(II) peak intensity at 710.0 eV showed a ~10% increase following repeated exposure to X-ray light, whereas the Fe(III) peak at 711.8 eV showed an ~10% decrease.

Since we generally limited the exposure of particles to mapping at 2- or 3-energies (for Fe L-edge, and 3 - 4 energies for the C K-edge) instead of entire energy range (e.g., 700 - 735 eV) required for a stack, we conclude that the impact of beam damage is negligible for the work conducted here.

Supplementary Note 4. Distribution of Fe within particles

The homogeneity/heterogeneity of Fe distribution within particles was evaluated using the total X-ray absorption at the Fe L-edge (Fe(II)+Fe(III)). This was done using the shape of the optical density profile across a longitudinal transect of the particle (shown as 'Pixel number'

in Supplementary Fig. 10 d/e/f), where the particles were assumed to be present as semi-circular spheres on the substrate surface.[8]

Fe was observed to be homogeneously distributed through particles – as determined by the semi-circular shape of the total Fe optical density profile - under high RH conditions in the STXM environmental cell (Supplementary Fig. 10 a/d and b/e) and not in dense localized regions, regardless of the relative humidity that the SOA were formed at. This suggests that Fe was well mixed in our samples under high RH conditions, even if some initial separation existed for samples formed in the atmospheric simulation chamber at low RH. Under low RH conditions (Supplementary Fig. 10 c/f) in the STXM environmental cell the profile of the total Fe optical density was less spherical in shape, with a greater slope near the edges of the particle. This suggests that the particles formed at low RH in the atmospheric simulation chamber and present in the STXM environmental cell under low RH conditions, exhibited some degree of phase separation. Although, there was still some Fe present near the particle perimeters, perhaps from slow diffusion of Fe and mixing of particles (at least to some degree) in between collection and analysis (~9 – 10 days).

Supplementary Note 5. STXM/NEXAFS analysis of Fe maps

Two major absorption peaks for Fe in our Fe-containing SOA samples were observed between an energy range of approximately 707 – 717 and 720 – 730 eV, corresponding to the L_3 $2_{p3/2}$ and L_2 $2_{p1/2}$ orbitals of the Fe L-edge respectively (Supplementary Fig. 14). The former was used to calculate the Fe oxidation state as either ferrous (Fe(II)) or ferric (Fe(III)) with peaks at 710.0 eV and 711.8 eV, respectively due to its greater signal intensity compared to the peaks for the L_2 $2_{p1/2}$ orbital. We observed a difference in the peak energies of 1.8 eV, which agrees with the difference of 1.7eV in peak absorption energies for Fe(II) (707.8 eV;

$FeCl_2$) and Fe(III) (709.5 eV; $FeCl_3$) that was reported by Moffet et al. 2012,[9] and work conducted by our group previously.[7, 8]

2- or 3-energy 'maps' were collected (e.g., Fig. 3a/b of the main manuscript) and used to determine the relative contribution of different Fe oxidation states (Fe(II) or Fe(III)) to the total Fe(II)+Fe(III) signal. These maps were collected by generating an image across a grid for the absorption at a specific energy, i.e., 710.0 eV for Fe(II) and 711.8 eV for Fe(III). For some samples, a 'pre-edge' image was not collected to save on acquisition time, which was useful for the maps conducted post-irradiation when we wanted to maximize time resolution. For the experiments where a 'pre-edge' image was not collected, a pre-edge based on a calculated parameter was used instead as described previously by Alpert et al. 2019.[8]

Supplementary Figures and Tables

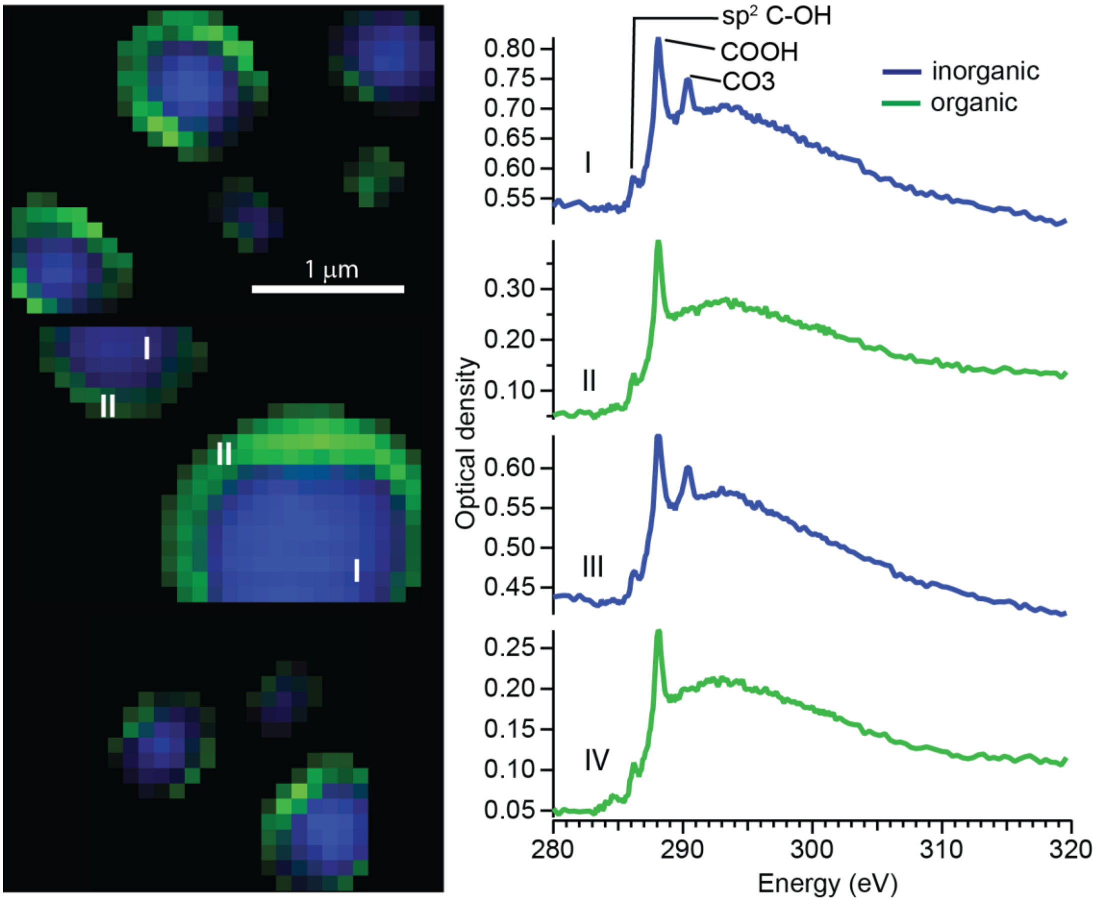

Supplementary Fig. 1. Carbon K-edge NEXAFS spectra and corresponding X-ray component images of particles collected on Cu TEM grids from the atmospheric simulation chamber for an experiment conducted at low RH (<10%). Spectra I and II, are from the 'inorganic' and 'organic' like, i.e., blue and green regions, labeled 'I' & 'II' of the corresponding X-ray component images. Spectra III and IV are the average spectra for all particles shown. The scale bar is 1 µm and applies to all images.

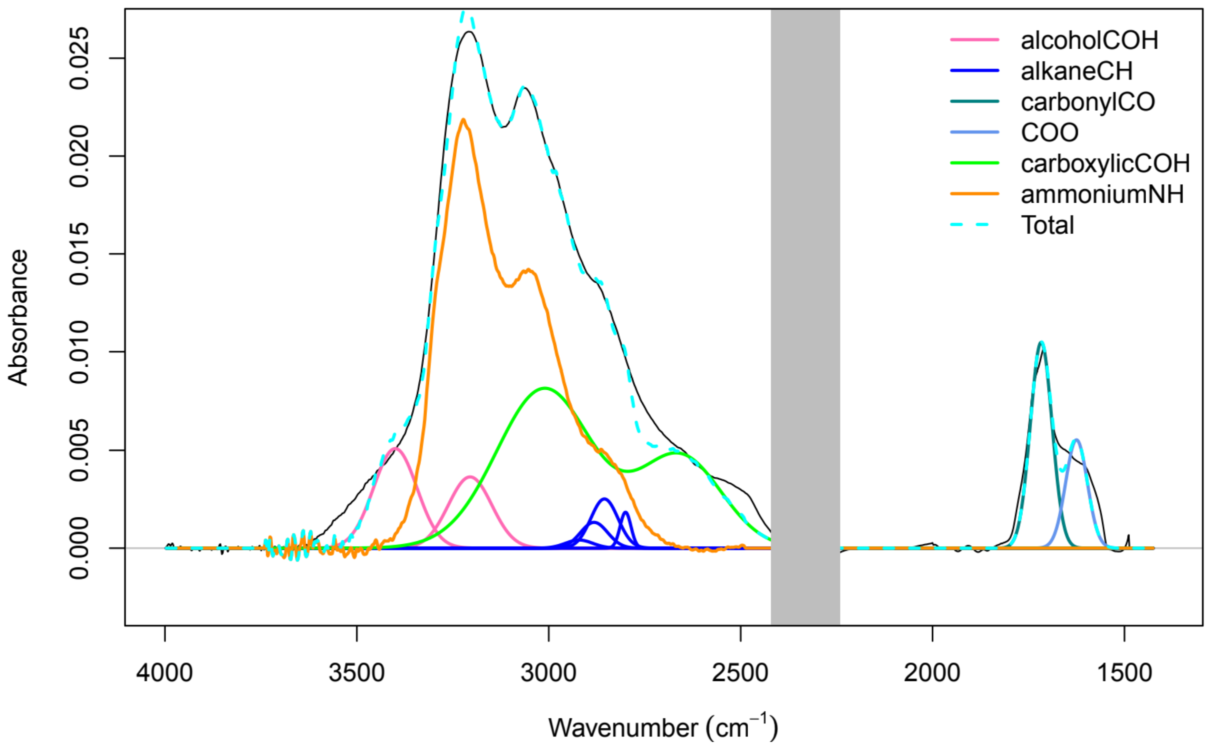

Supplementary Fig. 2. Sample FTIR spectrum (low RH, post-UV) showing peak assignment for different functional groups fit. The total signal is shown as the dashed line, and different stretches and their associated functional groups as the different coloured lines. The gray rectangle masks the region with interferences from carbon dioxide remaining in the sample compartment of the FTIR.

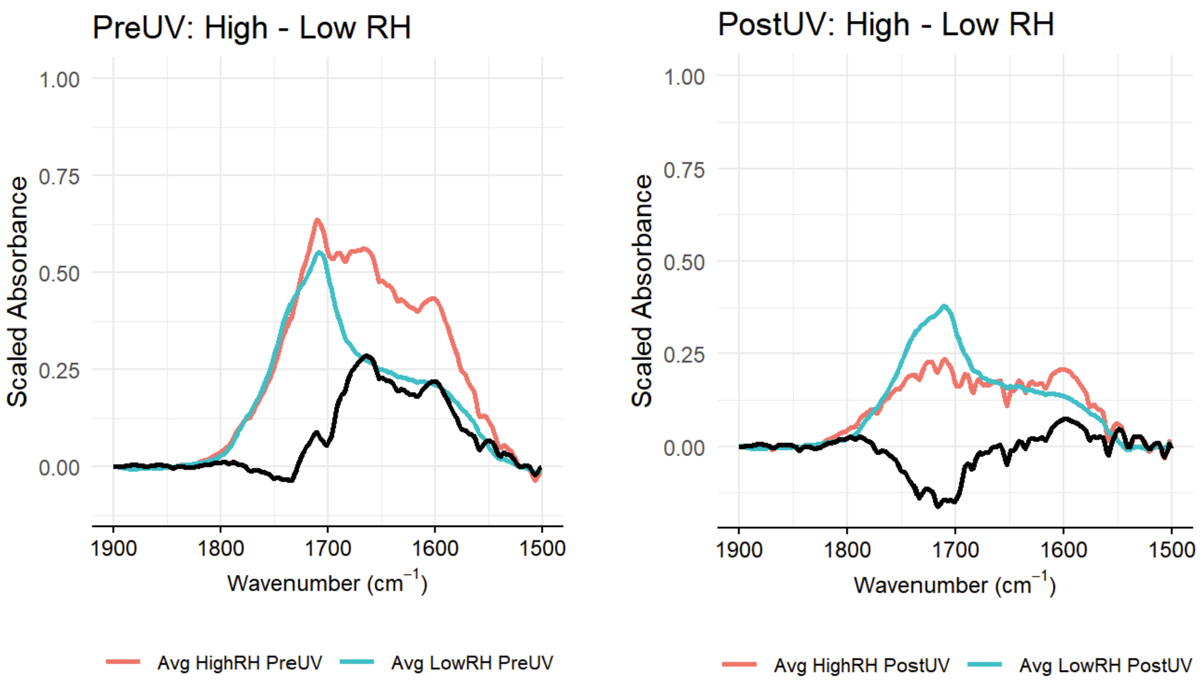

Supplementary Fig. 3. Sample FTIR spectra for Fe-containing SOA formed in the atmospheric simulation chamber at high (red trace) and low (teal trace) RH, collected pre-UV (left) and post-UV (right) exposure in the atmospheric simulation chamber. Spectra were normalized by their highest peak, replicates were averaged and the averages subtracted one to the other (High RH – Low RH). The difference between the spectra for SOA formed at high vs low RH are shown as the black line.

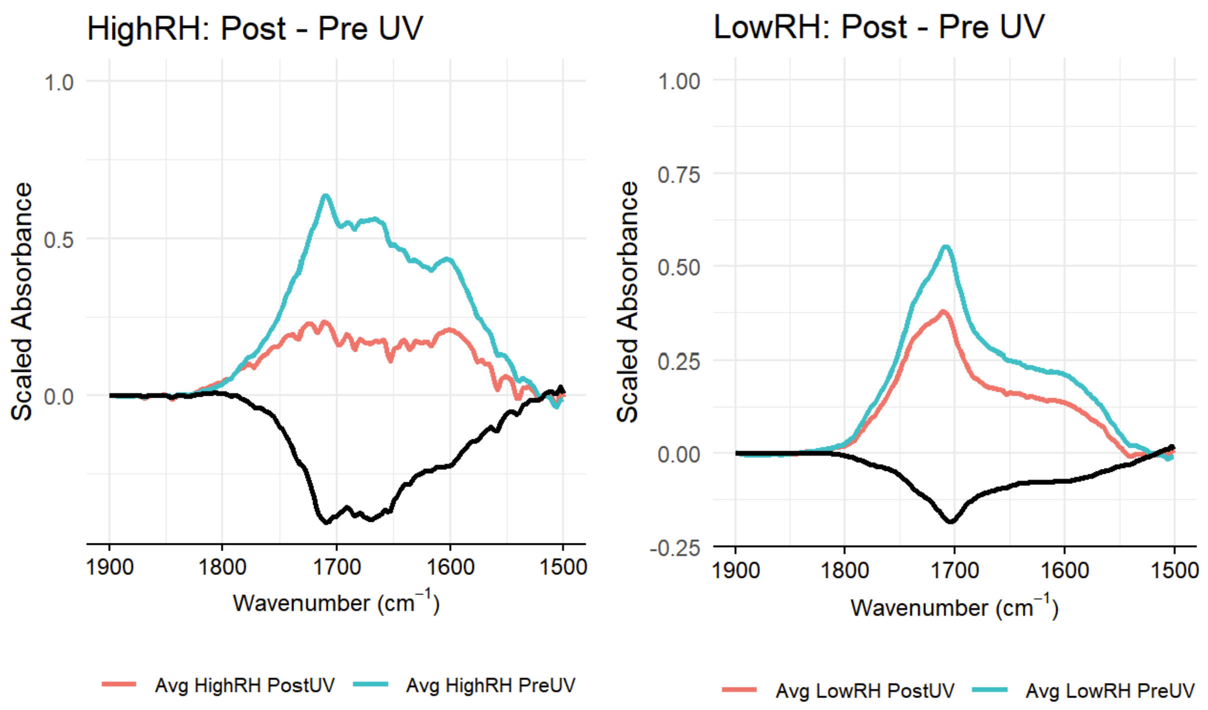

Supplementary Fig. 4. Sample FTIR spectra for Fe-containing SOA formed in the atmospheric simulation chamber at high (left) and low (right) RH. The red trace depicts the spectrum for SOA samples collected post-UV exposure and the teal trace depicts the spectrum for SOA samples collected pre-UV exposure. The difference between the spectra post and pre-UV exposure are shown as the black line.

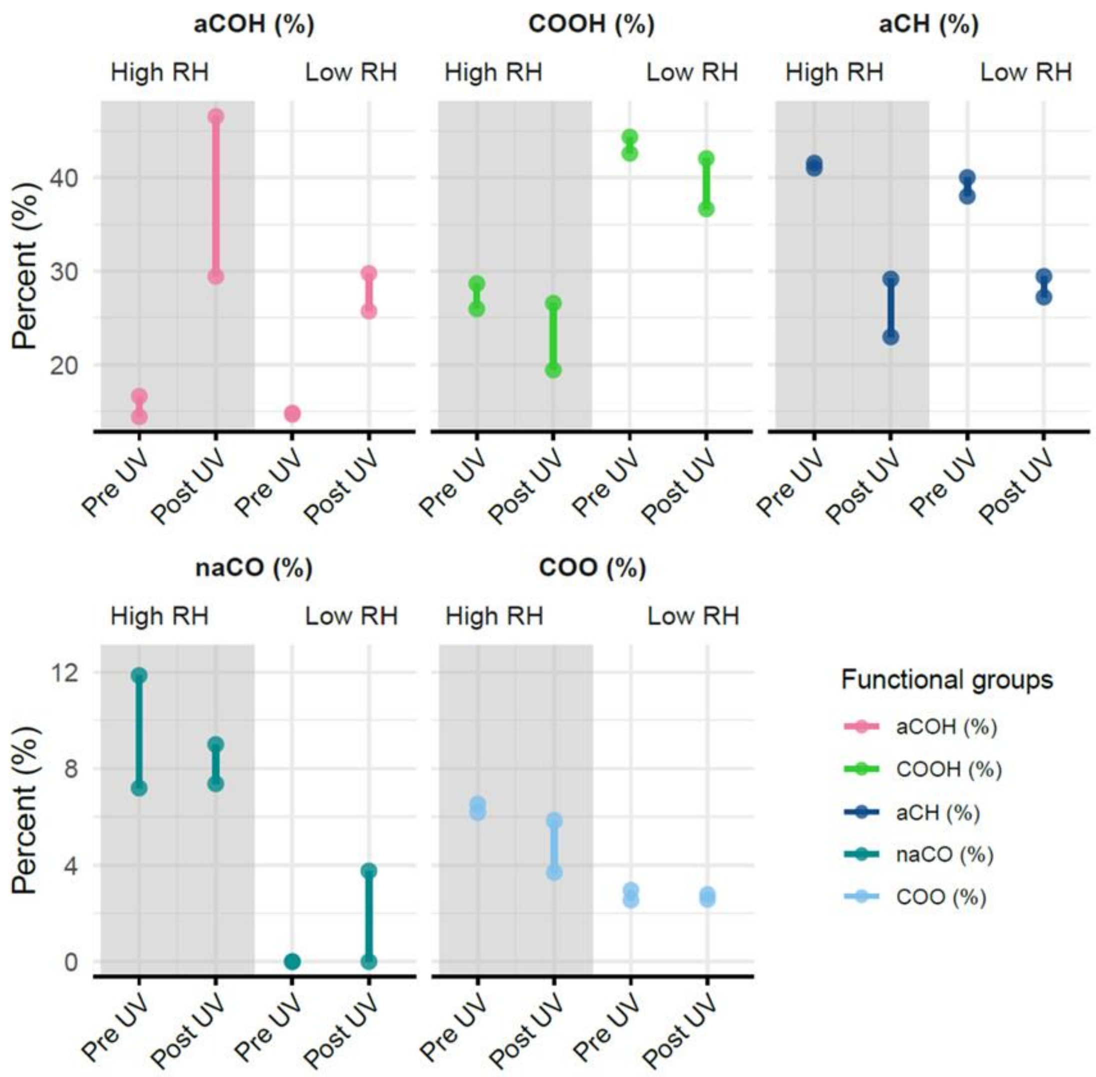

Supplementary Fig. 5. Variability of FTIR functional groups shown in Fig. 2 and Supplementary Table 2. The plot shows the variability of samples' functional groups under different relative humidity (RH) and UV exposure conditions. The shaded grey background highlights the high RH conditions for each functional group. Each point represents a sample, with lines connecting replicates of samples to highlight their variability. Pre-UV and Post-UV conditions are directly compared for the same functional group to show trends and magnitude in functional group variability due to UV exposure. aCOH = alcohol, COOH = carboxylic acid, aCH = alkane, naCO = non-acid carbonyl, COO = carboxylate.

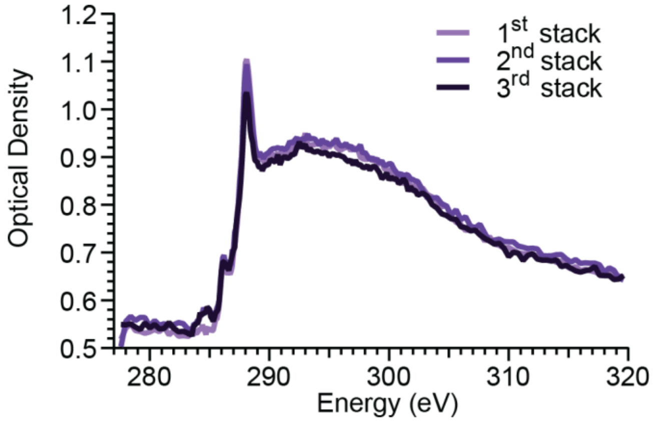

Supplementary Fig. 6. Beam damage was assessed by conducting replicate full near edge X-ray absorption fine structure (NEXAFS) spectra over the same particles, where each pixel was irradiated with approximately 1700 photons at 186 energy points between 277 – 320 eV. The consecutive spectra are shown as the different purple-coloured traces.

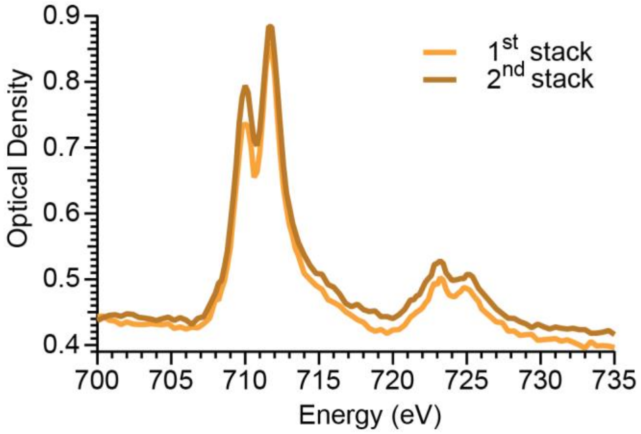

Supplementary Fig. 7. Beam damage was assessed by conducting replicate full NEXAFS spectra over the same particles, where each pixel was irradiated with approximately 1700 photons at 113 energy points between 700 – 735 eV. The consecutive spectra are shown as the different yellow- and brown-coloured traces.

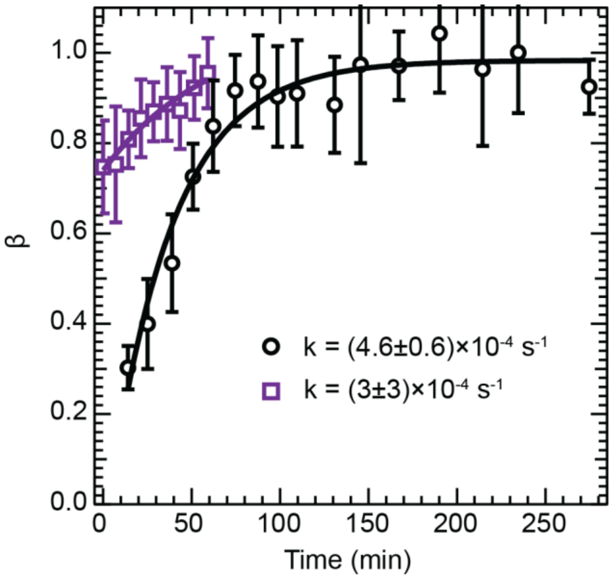

Supplementary Fig. 8. Reoxidation rates for Fe present in SOA formed at low (purple squares) and high RH (black circles) in the presence of ~300 ppb O_3. These rates correspond to the data shown in Fig. 3c/d of the main manuscript.

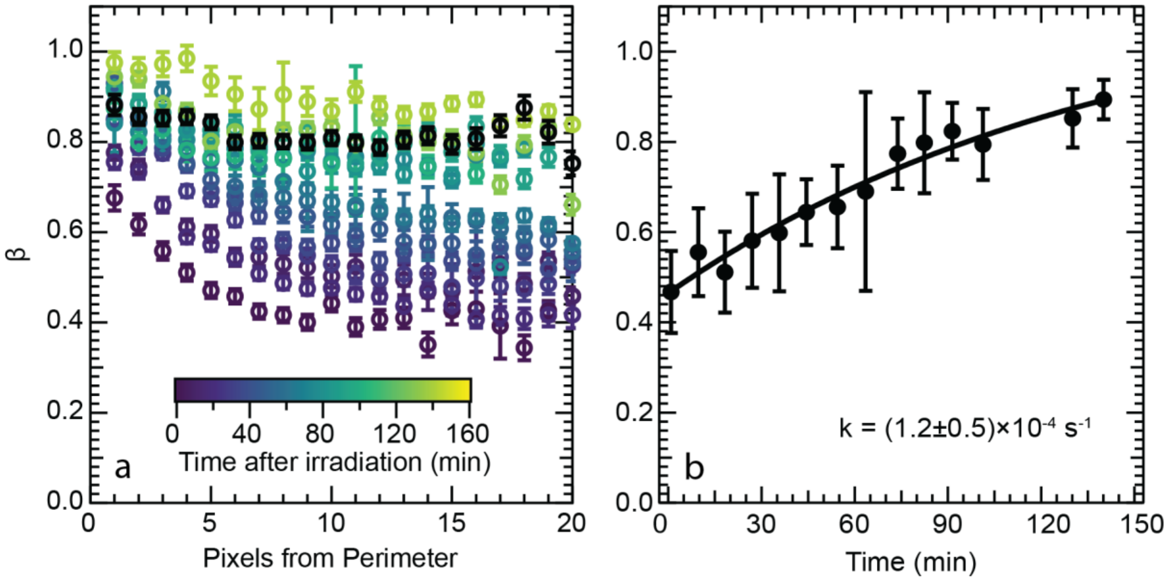

Supplementary Fig. 9. (A) Average fraction of Fe(III), β, for each 'pixel from perimeter' i.e., as a proxy distance from particle edge for SOA formed in the atmospheric simulation chamber at high RH and irradiated at 80% RH in the STXM environmental cell and exposed to O_3 (~20-30ppb). The data points are shown as the average β for individual time points (same colour) and the error bars are the 1 σ standard deviation. The black data points show the average β before irradiation. (B) Average β in SOA formed in the atmospheric simulation chamber at high RH and irradiated and exposure of particles to O_3 (~20-30ppb) in the STXM environmental cell at high RH (80%), as a function of time.

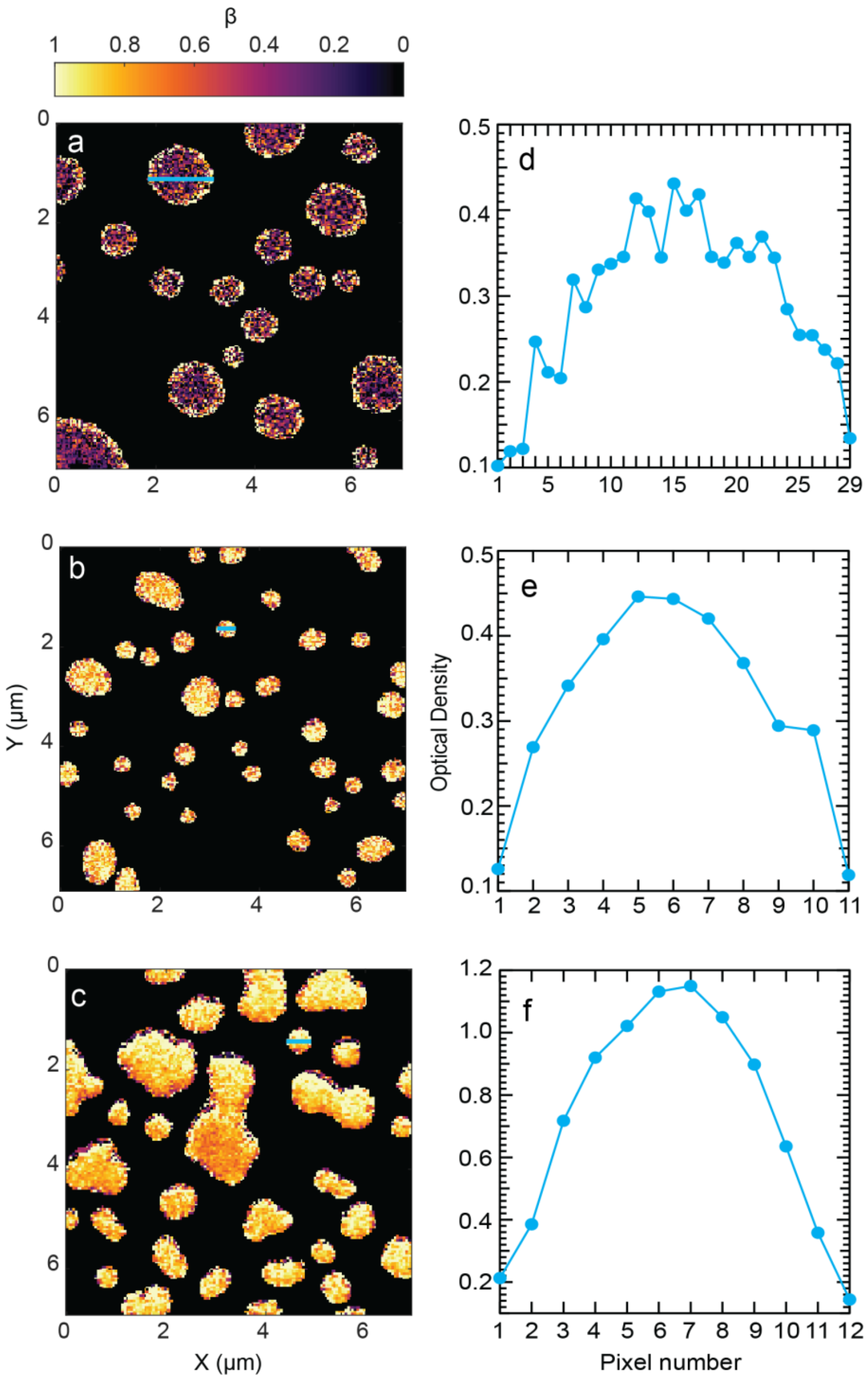

Supplementary Fig. 10. Sample Fe maps showing the Fe(III) fraction (β, colour scale) for particles generated in the atmospheric simulation chamber at (a) high and (b/c) low RH. (a/b) were maps taken for particles in the STXM environmental cell at high RH, and (c) for particles in the STXM environmental cell at low RH. (d/e/f) show the optical density due to total Fe (Fe(II)+Fe(III)) absorption across a cross section of particle, marked as a blue line in (a/b/c).

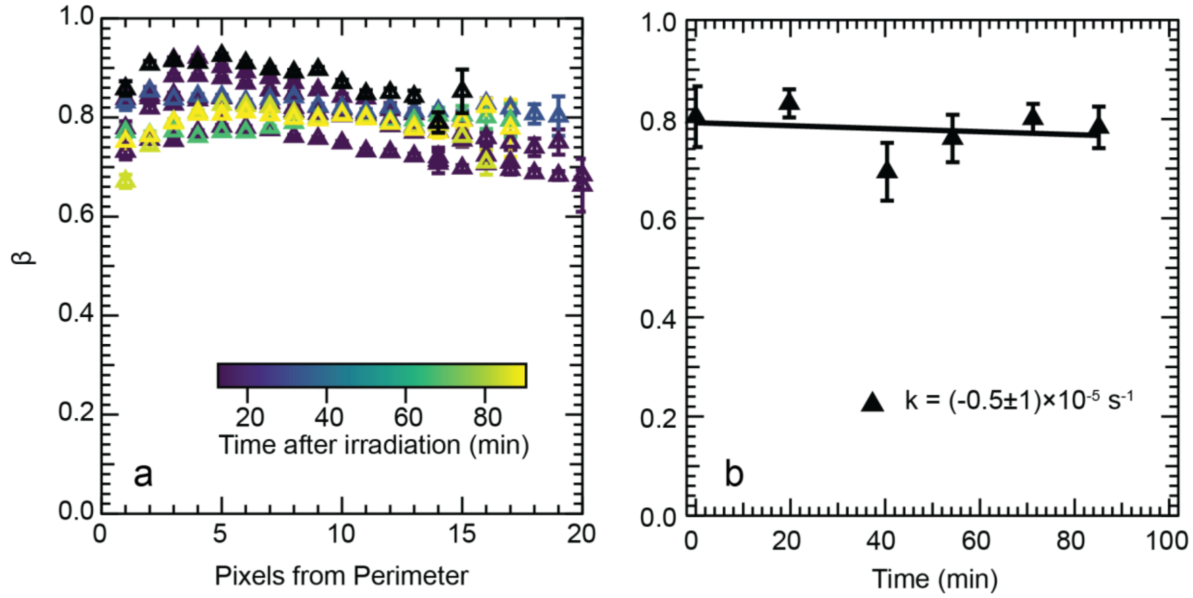

Supplementary Fig. 11. (A) Average fraction of Fe(III), β, for each 'pixel from perimeter' i.e., as a proxy distance from particle edge for SOA formed in the atmospheric simulation chamber at low RH and irradiated at 0% RH in the STXM environmental cell and exposed to O_2 (~80%). The data points are shown as the average β for individual time points (same colour) and the error bars are the 1 σ standard deviation. The black data points show the average β before irradiation. (B) Average β in SOA formed in the atmospheric simulation chamber at low RH and irradiated and exposure of particles to O_2 (80%) in the STXM environmental cell at low RH (0%), as a function of time.

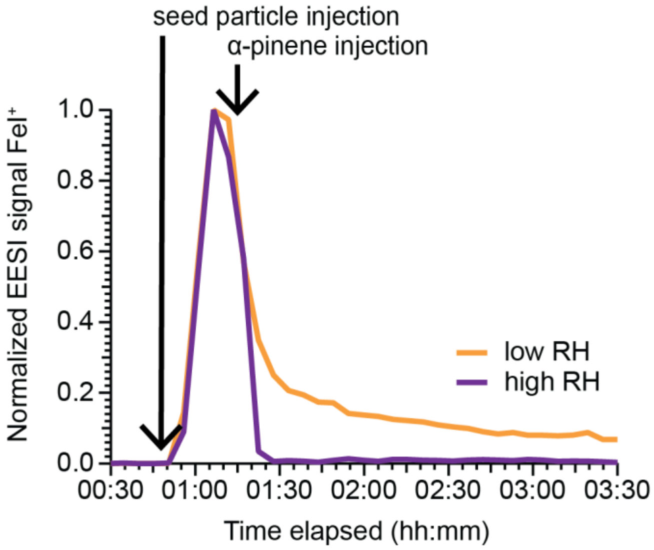

Supplementary Fig. 12. Timeseries of normalized extractive electrospray ionization mass spectrometry (EESI-MS) signal for the FeI^+ adduct for experiments conducted in the atmospheric simulation chamber at low (orange trace) and high (purple trace) RH.

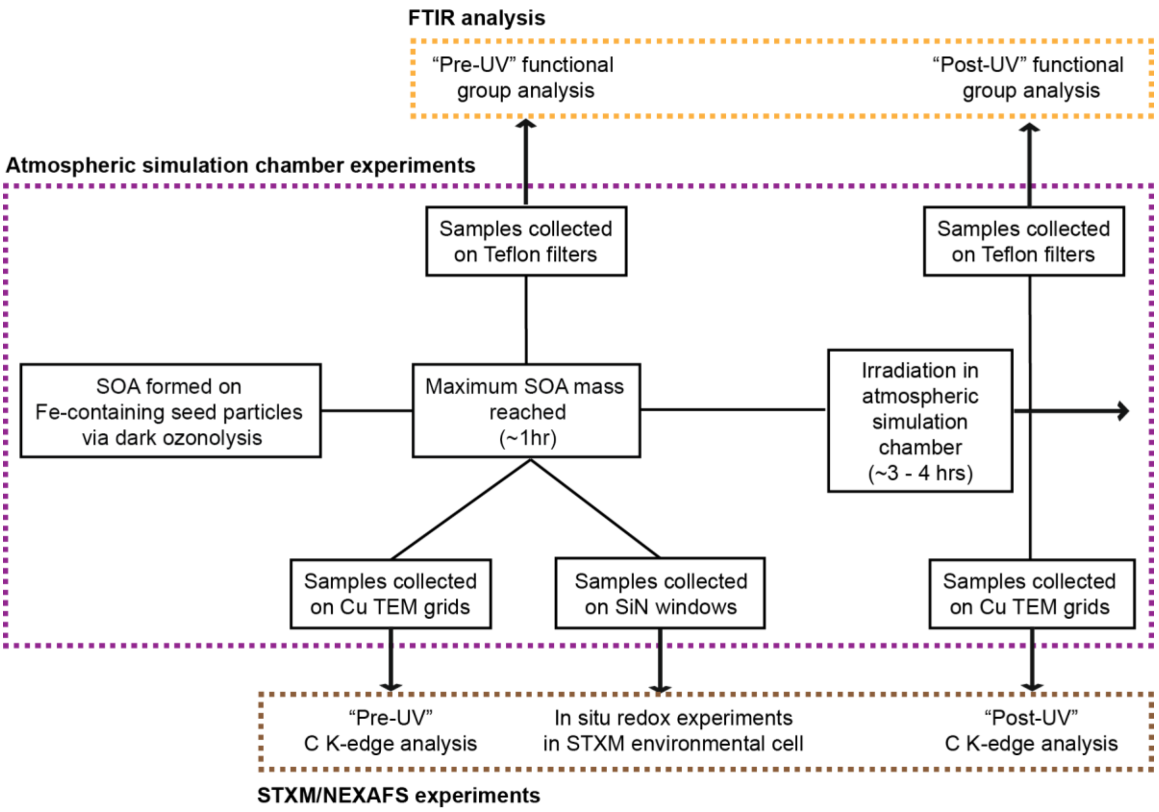

Supplementary Fig. 13. Flowchart showing the samples collected during the atmospheric simulation chamber experiments and their corresponding STXM/NEXAFS experiments. A summary of samples collected is shown in Supplementary Table 6.

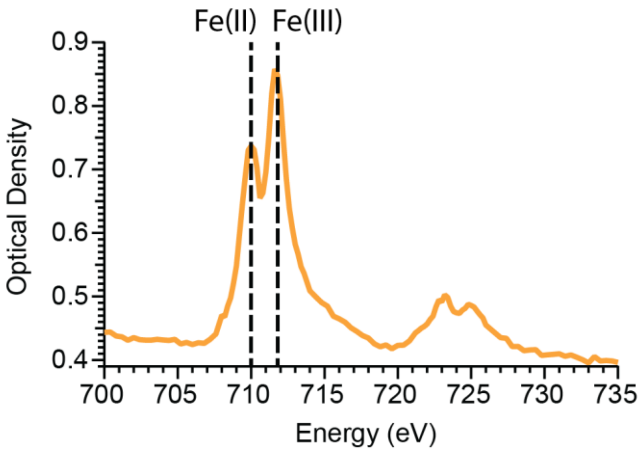

Supplementary Fig. 14. Sample NEXAFS spectrum at the Fe L-edges for Fe-containing SOA showing the absorption peaks for Fe(II) and Fe(III) at 710.0 and 711.8 eV, respectively.

Supplementary Table 1. Summary of the number of particles sampled for each carbon spectrum.

Spectrum #	Sample type	Number of particles
230508_089	Low RH, pre UV	1
230508_091	Low RH, pre UV	3
230508_093	Low RH, pre UV	1
230508_094	Low RH, pre UV	2
230507_114	High RH, pre UV	4
230507_117	High RH, pre UV	1
230507_118	High RH, pre UV	4
230507_119	High RH, pre UV	3
230507_153	Low RH, post UV	5
230507_154	Low RH, post UV	2
230507_155	Low RH, post UV	3
230507_156	Low RH, post UV	2
230506_098	High RH, post UV	1
230506_105	High RH, post UV	6
230506_112	High RH, post UV	4

Supplementary Table 2. Summary of FTIR functional group data shown in Fig. 2. The error on the average represents the 1σ standard deviation of the replicates. COOH = carboxylic acid, aCOH = alcohol, aCH = alkane, naCO = non-acid carbonyl, COO = carboxylate

**Sample**	**COOH (%)**	**aCOH (%)**	**aCH (%)**	**naCO (%)**	**COO (%)**
Low RH, pre-UV	43	15	40	0	3
Low RH, pre-UV	44	15	38	0	3
**Average**	**43 ± 1**	**15 ± 0**	**39 ± 1**	**0 ± 0**	**3 ± 0**
High RH, pre-UV	26	14	42	12	6
High RH, pre-UV	29	17	41	7	7
**Average**	**27 ± 2**	**16 ± 2**	**41 ± 0**	**10 ± 3**	**6 ± 0**
Low RH, post-UV	42	26	29	0	3
Low RH, post-UV	37	30	27	4	3
**Average**	**39 ± 4**	**28 ± 3**	**28 ± 2**	**2 ± 3**	**3 ± 0**
High RH, post-UV	19	47	23	7	4
High RH, post-UV	27	29	29	9	6
**Average**	**23 ± 5**	**38 ± 12**	**26 ± 4**	**8 ± 1**	**5 ± 2**

Supplementary Table 3. Summary of individual particle data for Fe(III) fraction of Fe-containing SOA shown in Fig. 3c.

**Sample #**	**SOA RH type**	**Particles/ map**	**Average β ± 1 σ**	**Maximum pixels/particle**	**Maximum particle radius (nm)**
20230623_041	High RH	23	0.8±0.1	16	560
20230623_097	High RH	19	0.30±0.05	17	595
20230623_100	High RH	22	0.4±0.1	21	735
20230623_103	High RH	21	0.5±0.1	26	910
20230623_106	High RH	25	0.73±0.07	21	735
20230623_109	High RH	16	0.8±0.1	30	1050
20230623_112	High RH	19	0.92±0.08	30	1050
20230623_115	High RH	24	0.9±0.1	24	840
20230623_118	High RH	19	0.9±0.1	25	875
20230623_121	High RH	21	0.9±0.1	24	840
20230623_127	High RH	25	0.9±0.1	24	840
20230623_132	High RH	16	1.0±0.2	40	1400

Supplementary Table 4. Summary of individual particle data for Fe(III) fraction of Fe-containing SOA shown in Fig. 3d.

**Sample #**	**SOA RH type**	**Particles/ map**	**Average β ± 1 σ**	**Maximum pixels/particle**	**Maximum particle radius (nm)**
20230624_021	Low RH	30	0.92±0.05	15	525
20230624_029	Low RH	29	0.7±0.7	21	735
20230624_032	Low RH	32	0.8±0.1	33	1155
20230624_035	Low RH	38	0.81±0.06	27	945
20230624_038	Low RH	34	0.86±0.09	41	1435
20230624_041	Low RH	30	0.87±0.07	24	840
20230624_044	Low RH	21	0.89±0.08	24	840
20230624_047	Low RH	25	0.87±0.09	41	1435
20230624_050	Low RH	35	0.92±0.07	13	455
20230624_054	Low RH	43	0.95±0.08	20	700

Supplementary Table 5. Photochemistry and stability constants data for selected Fe-inorganic and Fe-carboxylate complexes. L = ligand, ε = molar absorptivity

Complex	pH	Fe:L	ε around ~360 nm (M^{-1} cm^{-1})	Stability constant	Ref.
$Fe(OH)^{2+}$	2.5-4	1:1	355	-2.2	[10-13]
$Fe(SO_4)^{+}$	3	1:1	576	4.27	[11, 12, 14]
**Fe-unsubstituted ligands**					
oxalate	3-5	1:2	753 ± 357	23.66	[15]
malonate	4.25	1:2	210	13.54	[11]
succinate	3.5	1:2	842±92	13.34	[11]
glutarate	4	1:2	1134	13.12	[11]
**Fe-OH substituted ligands**					
citrate	5.5	1:1	796	25.69	[16-18]
tartrate	4.5	1:2	1548±13	10.53	[11]
gluconate	8	1:2(3OH)	1638±13	22.23	[11]
lactate	3	1:1	1374	-1.56	[11, 19]
malate	3	1:2	200	15.21	[11, 20]
**Fe-keto/diol substituted ligands**					
pyruvate	3	1:1	1160	4.83	[11]
glyoxalate	3	1:1	1001	13.9	[11]

Supplementary Table 6. Summary of samples collected.

Sample #	RH	UV exposure (Pre/Post)	Substrate type	Analysis method
20230502_1155	Low	Pre UV	Cu TEM grid	STXM/NEXAFS, Under vacuum @ C K-edge
20230502_1407	Low	Post UV	Cu TEM grid	STXM/NEXAFS, Under vacuum @ C K-edge
20230503_1130	High	Pre UV	Cu TEM grid	STXM/NEXAFS, Under vacuum @ C K-edge
20230503_1326	High	Post UV	Cu TEM grid	STXM/NEXAFS, Under vacuum @ C K-edge
15	Low	Pre UV	SiN window	STXM/NEXAFS, environmental cell
4	Low	Pre UV	SiN window	STXM/NEXAFS, environmental cell
20	High	Pre UV	SiN window	STXM/NEXAFS, environmental cell
14	High	Pre UV	SiN window	STXM/NEXAFS, environmental cell
20230613_1412	Low	Pre UV	Teflon filter	FTIR
20230613_1413	Low	Pre UV	Teflon filter	FTIR
20230613_1701	Low	Post UV	Teflon filter	FTIR
20230613_1702	Low	Post UV	Teflon filter	FTIR
20230614_1113	High	Pre UV	Teflon filter	FTIR
20230614_1114	High	Pre UV	Teflon filter	FTIR
20230614_1346	High	Post UV	Teflon filter	FTIR
20230614_1347	High	Post UV	Teflon filter	FTIR

Supplementary References

(1) Reggente, M.; Höhn, R.; Takahama, S. An open platform for Aerosol InfraRed Spectroscopy analysis – AIRSpec. *Atmos. Meas. Tech.* **2019**, *12* (4), 2313-2329. DOI: 10.5194/amt-12-2313-2019.

(2) Russell, L. M.; Bahadur, R.; Ziemann, P. J. Identifying organic aerosol sources by comparing functional group composition in chamber and atmospheric particles. *Proc. Natl. Acad. Sci. U.S.A.* **2011**, *108* (9), 3516-3521. DOI: doi:10.1073/pnas.1006461108.

(3) Reggente, M.; Dillner, A. M.; Takahama, S. Analysis of functional groups in atmospheric aerosols by infrared spectroscopy: systematic intercomparison of calibration methods for US measurement network samples. *Atmos. Meas. Tech.* **2019**, *12* (4), 2287-2312. DOI: 10.5194/amt-12-2287-2019.

(4) Takahama, S.; Johnson, A.; Russell, L. M. Quantification of Carboxylic and Carbonyl Functional Groups in Organic Aerosol Infrared Absorbance Spectra. *Aerosol Sci. Technol.* **2013**, *47* (3), 310-325. DOI: 10.1080/02786826.2012.752065.

(5) Wang, Y.; Liu, P.; Li, Y. J.; Bateman, A. P.; Martin, S. T.; Hung, H.-M. The Reactivity of Toluene-Derived Secondary Organic Material with Ammonia and the Influence of Water Vapor. *J. Phys. Chem. A* **2018**, *122* (38), 7739-7747. DOI: 10.1021/acs.jpca.8b06685.

(6) Abrahamson, H. B.; Rezvani, A. B.; Brushmiller, J. G. Photochemical and spectroscopic studies of complexes, of iron(III) with citric acid and other carboxylic acids. *Inorg. Chim. Acta* **1994**, *226* (1), 117-127. DOI: 10.1016/0020-1693(94)04077-X.

(7) Dou, J.; Alpert, P. A.; Corral Arroyo, P.; Luo, B.; Schneider, F.; Xto, J.; Huthwelker, T.; Borca, C. N.; Henzler, K. D.; Raabe, J.; et al. Photochemical degradation of iron(III) citrate/citric acid aerosol quantified with the combination of three complementary experimental techniques and a kinetic process model. *Atmos. Chem. Phys.* **2021**, *21* (1), 315-338. DOI: 10.5194/acp-21-315-2021.

(8) Alpert, P. A.; Corral Arroyo, P.; Dou, J.; Krieger, U. K.; Steimer, S. S.; Forster, J. D.; Ditas, F.; Pohlker, C.; Rossignol, S.; Passananti, M.; et al. Visualizing reaction and diffusion in xanthan gum aerosol particles exposed to ozone. *Phys. Chem. Chem. Phys.* **2019**, *21* (37), 20613-20627. DOI: 10.1039/c9cp03731d.

(9) Moffet, R. C.; Furutani, H.; Rödel, T. C.; Henn, T. R.; Sprau, P. O.; Laskin, A.; Uematsu, M.; Gilles, M. K. Iron speciation and mixing in single aerosol particles from the Asian continental outflow. *J. Geophys. Res.-Atmos.* **2012**, *117* (D7), D07204. DOI: 10.1029/2011jd016746.

(10) Faust, B. C.; Hoigné, J. Photolysis of Fe (III)-hydroxy complexes as sources of OH radicals in clouds, fog and rain. *Atmos. Environm. A* **1990**, *24* (1), 79-89. DOI: 10.1016/0960-1686(90)90443-Q.

(11) Weller, C.; Horn, S.; Herrmann, H. Photolysis of Fe(III) carboxylato complexes: Fe(II) quantum yields and reaction mechanisms. *J. Photochem. Photobiol. A* **2013**, *268*, 24-36. DOI: 10.1016/j.jphotochem.2013.06.022.

(12) Zhu, X.; Prospero, J. M.; Millero, F. J.; Savoie, D. L.; Brass, G. W. The solubility of ferric ion in marine mineral aerosol solutions at ambient relative humidities. *Mar. Chem.* **1992**, *38* (1), 91-107. DOI: 10.1016/0304-4203(92)90069-M.

(13) Feng, W.; Nansheng, D. Photochemistry of hydrolytic iron (III) species and photoinduced degradation of organic compounds. A minireview. *Chemosphere* **2000**, *41* (8), 1137-1147. DOI: 10.1016/S0045-6535(00)00024-2.

(14) Benkelberg, H.-J.; Warneck, P. Photodecomposition of Iron(III) Hydroxo and Sulfato Complexes in Aqueous Solution: Wavelength Dependence of OH and SO4- Quantum Yields. *J. Phys. Chem.* **1995**, *99*, 5214-5221. DOI: 10.1021/j100014a049.

(15) Weller, C.; Horn, S.; Herrmann, H. Effects of Fe(III)-concentration, speciation, excitation-wavelength and light intensity on the quantum yield of iron(III)-oxalato complex

photolysis. *J. Photochem. Photobiol. A* **2013**, *255*, 41-49. DOI: 10.1016/j.jphotochem.2013.01.014.

(16) Vukosav, P.; Mlakar, M.; Tomišić, V. Revision of iron(III)–citrate speciation in aqueous solution. Voltammetric and spectrophotometric studies. *Anal. Chim. Acta* **2012**, *745*, 85-91. DOI: 10.1016/j.aca.2012.07.036.

(17) Pozdnyakov, I. P.; Kel, O. V.; Plyusnin, V. F.; Grivin, V. P.; Bazhin, N. M. New Insight into Photochemistry of Ferrioxalate. *J. Phys. Chem. A* **2008**, *112* (36), 8316-8322. DOI: 10.1021/jp8040583.

(18) Alpert, P. A.; Dou, J.; Corral Arroyo, P.; Schneider, F.; Xto, J.; Luo, B.; Peter, T.; Huthwelker, T.; Borca, C. N.; Henzler, K. D.; et al. Photolytic radical persistence due to anoxia in viscous aerosol particles. *Nat. Commun.* **2021**, *12* (1), 1769. DOI: 10.1038/s41467-021-21913-x.

(19) Portanova, R.; Lajunen, L. H. J.; Tolazzi, M.; Piispanen, J. Critical evaluation of stability constants for α-hydroxycarboxylic acid complexes with protons and metal ions and the accompanying enthalpy changes **Part II: Aliphatic 2-hydroxycarboxylic acids***; 4; International Union of Pure and Applied Chemistry, 2003.

(20) Vukosav, P.; Tomišić, V.; Mlakar, M. Iron(III)-Complexes Engaged in the Biochemical Processes in Seawater. II. Voltammetry of Fe(III)-Malate Complexes in Model Aqueous Solution. *Electroanalysis* **2010**, *22* (19), 2179-2186. DOI: 10.1002/elan.200900632.
